# Release of cell-free enzymes by marine pelagic fungal strains

**DOI:** 10.3389/ffunb.2023.1209265

**Published:** 2023-11-06

**Authors:** Katherine Salazar-Alekseyeva, Gerhard J. Herndl, Federico Baltar

**Affiliations:** ^1^ Department of Functional and Evolutionary Ecology, Bio-Oceanography and Marine Biology Unit, University of Vienna, Vienna, Austria; ^2^ Department of Agrotechnology and Food Sciences, Bioprocess Engineering Group, Wageningen University and Research, Wageningen, Netherlands; ^3^ Department of Marine Microbiology and Biogeochemistry, Royal Netherlands Institute for Sea Research (NIOZ), University of Utrecht, Texel, Netherlands

**Keywords:** marine fungi, cell-free enzymatic activity, kinetics, maximum velocity, half-saturation constant

## Abstract

Fungi are ubiquitous organisms that secrete different enzymes to cleave large molecules into smaller ones so that can then be assimilated. Recent studies suggest that fungi are also present in the oceanic water column harboring the enzymatic repertoire necessary to cleave carbohydrates and proteins. In marine prokaryotes, the cell-free fraction is an important contributor to the oceanic extracellular enzymatic activities (EEAs), but the release of cell-free enzymes by marine fungi remains unknown. Here, to study the cell-free enzymatic activities of marine fungi and the potential influence of salinity on them, five strains of marine fungi that belong to the most abundant pelagic phyla (Ascomycota and Basidiomycota), were grown under non-saline and saline conditions (0 g/L and 35 g/L, respectively). The biomass was separated from the medium by filtration (0.2 μm), and the filtrate was used to perform fluorogenic enzymatic assays with substrate analogues of carbohydrates, lipids, organic phosphorus, sulfur moieties, and proteins. Kinetic parameters such as maximum velocity (V_max_) and half-saturation constant (K_m_) were obtained. The species studied were able to release cell-free enzymes, and this represented up to 85.1% of the respective total EEA. However, this differed between species and enzymes, with some of the highest contributions being found in those with low total EEA, with some exceptions. This suggests that some of these contributions to the enzymatic pool might be minimal compared to those with higher total EEA. Generally, in the saline medium, the release of cell-free enzymes degrading carbohydrates was reduced compared to the non-saline medium, but those degrading lipids and sulfur moieties were increased. For the remaining substrates, there was not a clear influence of the salinity. Taken together, our results suggest that marine fungi are potential contributors to the oceanic dissolved (i.e., cell-free) enzymatic pool. Our results also suggest that, under salinity changes, a potential effect of global warming, the hydrolysis of organic matter by marine fungal cell-free enzymes might be affected and hence, their potential contribution to the oceanic biogeochemical cycles.

## 1 Introduction

Low-molecular-weight (LMW) molecules are operationally defined as molecules with a molecular weight below 1000 Da, whereas high-molecular-weight (HMW) molecules compromise those larger than 1000 Da (Benner et al., 1992; Amon and Benner, 1996; Benner, 2002). In marine ecosystems, the majority of dissolved organic matter (DOM) is composed of LMW molecules, but the bioavailable ones are mainly HMW molecules (Wheeler, 1976; Amon and Benner, 1994; Amon and Benner, 1996; Benner, 2002). In order to take them up, microorganisms need to hydrolyze them into smaller molecules (<600 Da) (Weiss et al., 1991).

Osmotrophy is a feeding strategy that involves the secretion of different enzymes to transform large molecules into smaller ones which can then be absorbed by osmosis (Richards et al., 2012; Muszewska et al., 2017). For the fungal kingdom, osmotrophy is a distinctive feature that has allowed them to use largely inaccessible nutrients and conquer diverse environments (Dix and Webster, 1995; Webster and Weber, 2007; Richards and Talbot, 2013), including marine ones. Here, the fungal species can be obligate or facultative (Jennings, 1983). Obligate marine fungi are only capable of living in marine environments, while facultative species have a terrestrial origin, but are capable of also living in them (Raghukumar, 2017).

Total extracellular enzymatic activities (EEAs) are a combination of cell-attached and cell-free enzymatic activities (Wetzel, 1991; Baltar, 2018). Operationally defined, cell-attached enzymes are those retained on a 0.2 μm filter, whereas cell-free enzymes pass this filter (Baltar, 2018). In marine environments, the first ones are linked to the cell wall or periplasmic space, whereas cell-free enzymes are dissolved in the immediate waters (Hoppe et al., 2002). As cell-attached enzymes are tightly linked to the cell, and respond to substrates outside the cell, they represent a chemical communication with the surrounding environment (Chróst, 1990). In contrast, cell-free enzymes are released by the cells into the surrounding environment (Priest, 1977; Chróst, 1990; Wetzel, 1991). As these are not linked anymore to the cell, they are not metabolically controlled by the cell (Kamer and Rassoulzadegan, 1995). However, in the case of substrate limitation, cell-free enzymes can be used as a strategy “to find food fast” (Chandrasekaran and Kumar, 1997), and also to utilize other polymeric compounds which are otherwise non-usable (Chróst, 1990; Chróst, 1992). The occurrence of cell-free enzymes in marine environments might be crucial as these can access distant substrates, and influence the kinetics of organic matter (Kamer and Rassoulzadegan, 1995). This mobilization might also result in an improvement in substrate availability (Wetzel, 1991). As coined by Baltar (2018), cell-free enzymes are a kind of “living dead”, as they are not attached anymore to the cell, but can still perform their respective function. Moreover, as suggested by Arnosti (2011), cell-free enzymes can influence the carbon cycle at different times and spaces where they were originally produced. Therefore, a dissociation between marine microorganisms and enzymatic activities might exist (Arnosti, 2011; D’ambrosio et al., 2014; Baltar et al., 2016; Muszewska et al., 2017; Thomson et al., 2019).

Marine microbial EEAs contribute significantly to the breakdown of organic substrates (Hoppe et al., 2002). The majority of them were originally believed to be cell-attached rather than cell-free (Hoppe, 1983; Rego et al., 1985; Chróst, 1990; Chróst and Rai, 1993; Hoppe et al., 2002). Contrary, other studies suggested that the cell-free EEAs can be similar to or even higher than the cell-attached enzymatic pool (Kamer and Rassoulzadegan, 1995; Keith and Arnosti, 2001; Baltar et al., 2010; Duhamel et al., 2010; Allison et al., 2012; Baltar et al., 2013; Baltar et al., 2016; Baltar, 2018). Additionally, some studies pointed out that the sources of marine cell-free enzymes can be numerous (Kamer and Rassoulzadegan, 1995; Allison et al., 2012; Baltar et al., 2013), but believed to be mostly of bacterial origin (Hollibaugh and Azam, 1983; Chróst, 1990; Hoppe and Ullrich, 1999; Obayashi and Suzuki, 2008b; Baltar et al., 2010; Bong et al., 2013; D’ambrosio et al., 2014; Li et al., 2019). However, in a study on the upwelling ecosystem of Chile, Gutiérrez et al. (2011) found potential evidence of an active contribution of marine fungi to the total enzymatic pool, suggesting that they could also be involved in the breakdown of organic matter in the ocean.

As shown by Vetter and Deming (1999), cell-free enzymes act at a distance from the microorganism that originally released them, and in the absence of dissolved organic matter, these enzymes used particulate organic matter which provided enough hydrolysate to support microbial growth. Therefore, cell-free enzymes might be secreted to increase the chance of survival of the cell that originally produced them (Chróst, 1986; Chróst and Overbeck, 1987). But compared to the cell-attached enzymes, cell-free enzymes might also benefit other cells of the surrounding environment (Luo et al., 2009).

Due to anthropogenic and natural causes, salinity fluctuations have been reported in different oceanic regions (Skliris et al., 2014). These changes can lead microorganisms to experience osmotic and ionic stress (Gladfelter et al., 2019), and influence their extracellular enzymatic activities (Chróst, 1990; Caruso and Zaccone, 2000; Salazar-Alekseyeva et al., 2022)[in revision]). Marine fungi seem to tolerate a wide range of salinities (Jennings, 1983), but they are probably not halophilic (Gladfelter et al., 2019). Hence, it is currently unknown how changes in oceanic salinities might affect fungi and their EEAs, especially, the cell-free fraction.

Compared to bacteria, marine fungi are less studied, so here we investigated their secretion of cell-free enzymes using five species as representatives of the most dominant marine pelagic fungal phyla: Ascomycota, and Basidiomycota (Taylor and Cunliffe, 2016; Amend et al., 2019; Morales et al., 2019). These species were grown in non-saline and saline media to resemble conditions of freshwater and marine environments, respectively. The cell-free fraction EEA was determined and the potential effect of salinity on the kinetic parameters such as maximum velocity (V_max_) and half-saturation constant (K_m_) was analyzed.

## 2 Methods

The marine fungal species *Blastobotrys parvus* (HA 1620), *Metschnikowia australis* (HA 635), *Rhodotorula sphaerocarpa* (HB 738), and *Sakaguchia dacryoidea* (HB 877) were obtained from the Austrian Center of Biological Resources (ACBR), but were originally isolated from the Antarctic Ocean (Fell and Hunter, 1968; Newell and Fell, 1970; Fell and Statzell, 1971; Fell et al., 1973). In the case of *Rhodotorula mucilaginosa*, this was isolated from the Atlantic Ocean during the Poseidon Cruise in March 2019. All these pure isolates were maintained on yeast malt extract agar (Wickerham, 1939; Wickerham, 1951) at room temperature, and were renewed monthly.

To culture these species, the protocols of Salazar Alekseyeva et al. (2021) and Salazar-Alekseyeva et al. (2023) [in revision] were followed. One medium containing 2 g/L of glucose, malt extract, peptone, yeast extract, and 0.5 g/L of chloramphenicol was prepared and further divided into two media. The first one contained 35 g/L of artificial sea salts (S9883 Sigma-Aldrich), and the second one did not contain salts (0 g/L). In a vertical laminar airflow cabinet (Steril Bio Ban 72), with a sterile loop, an arbitrary amount of one-week-old pure isolates was transferred into autoclaved artificial seawater (35 g/L sea salts S9883 Sigma-Aldrich) until an optical density of ≈ 1 at 660 nm wavelength (OD_660_) was reached. This was measured with a UV-1800 Shimadzu spectrophotometer and represented a correlation with the cell count. Per every 1 L of the autoclaved medium, 0.01 L of this fungal dilution was added. Afterwards, 150 mL of this mixture (medium and fungal dilution) were put in Schott bottles and incubated at 5°C on a rotary shaker (Jeio Tech ISS-7100 Incubated Shaker) until the exponential phase was reached. Three bottles with similar OD_660_ values (biological triplicates) were chosen for further analyses (EEAs and biomass).

To estimate the fungal biomass that was releasing cell-free enzymes, 40 mL of the sample was vacuum filtered onto a pre-weighed and combusted (450°C for 6 h) Whatman GF/F filter (WHA1825047 Sigma-Aldrich, 47 mm diameter), and for 3 days, this filter was dried at 80°C. Finally, the filter was weighed again, and the fungal biomass was estimated from the difference between the pre-weighted filter and the dried one.

To estimate the fungal abundance that was releasing cell-free enzymes, 1.5 mL of the liquid media containing fungi was used. For a single-cell suspension, this volume was filtered onto a pluriStrainer Mini (43-10040-50 pluriSelect, 40 µm mesh size), fixed with a final concentration of 0.5% glutaraldehyde for 10 minutes, and lastly frozen at −80°C until further processing. As *B. parvus* has a filamentous structure, its abundance was not possible to estimate with this method. For the other species, depending on the OD_660_ value, 10 to 40 μL of the thawed sample and 5 μL of SYBR^®^ Green 100x (S9430, Sigma-Aldrich) were added, and completed with Tris EDTA buffer (TE) to obtain a final volume of 500 μL. Finally, the sample was measured with a BD Accuri™ C6 Plus Flow Cytometry set at ‘Run with limits’ of 10,000 events and ‘Medium’, and the cell abundance was estimated with the BD Accuri C6 Software.

To determine the cell-specific biomass, 1.5 mL of sample were filtered with a pluriStrainer Mini (43-10040-50 pluriSelect, 40 µm mesh size) to obtain a single-cell suspension. The sample was fixed in the dark with 0.5% (final conc.) glutaraldehyde for 10 minutes, and subsequently frozen at −80°C until further processing. Due to the multicellular structures of the filamentous species *B. parvus*, its cell abundance could not be determined. For the other species, depending on the optical density, 10 to 40 μL of the sample was diluted with TE to obtain a final volume of 500 μL which was later stained with 5 μL SYBR^®^ Green 100x (S9430, Sigma-Aldrich). The cell abundance was determined using a BD Accuri™ C6 Plus Flow Cytometry set at ‘Run with limits’ of 10,000 events and ‘Medium’ and the cell abundance was obtained with the BD Accuri C6 Software.

To obtain the cell-free fraction, the protocols of Hollibaugh and Azam (1983) and Kim et al. (2007) together with the suggestion of Obayashi and Suzuki (2008a) were followed. This was obtained by separating the biomass through vacuum filtration on Whatman Track-Etched Membranes with a pore size of 0.22 μm (WHA10417012 Sigma-Aldrich, 47 mm diameter). To maintain the integrity of the cells, the pressure did not exceed 100 mbar (Karner and Herndl, 1992).

To estimate the enzymatic activity, the protocols of Hoppe (1983); Salazar Alekseyeva et al. (2022), and Salazar-Alekseyeva et al. (2023) [in revision] were followed. The fluorogenic substrate analogues 4-methylumbelliferyl β-D-glucopyranoside (M3633 Sigma-Aldrich), 4-methylumbelliferyl β-D-xylopyranoside (M7008 Sigma-Aldrich), 4-methylumbelliferyl *N*-acetyl-β-D-glucosaminide (M2133 Sigma-Aldrich), 4-methylumbelliferyl-oleate (75164 Sigma-Aldrich), 4-methylumbelliferyl phosphate (M8883 Sigma-Aldrich), 4-methylumbelliferyl sulfate potassium salt (M7133 Sigma-Aldrich), N-succinyl-Ala-Ala-Pro-Phe-7-amido-4-methylcoumarin (L2145 Sigma-Aldrich), and t-butyloxycarbonyl-L-phenylalanyl-L-seryl-L-arginine-7-amido-4-methylcoumarin (3107-v PeptaNova) were used to determine the potential activity of the enzymes β-glucosidase (BGL), β-xylosidase (BXY), *N*-acetyl-β-D-glucosaminidase (NAG), lipase (OLE), alkaline phosphatase (APA), sulfatase (SUL), leucine aminopeptidase (LAP), and trypsin (TRY), respectively (**Table 1**). According to the targeted substrate, the enzymes were classified as cleaving carbohydrates (BGL, BXY, and NAG); lipids, phosphorus and sulfur moieties (OLE, APA, and SUL, respectively), and proteins (LAP and TRY). Consistently, methylcoumaryl amide (MCA) (A9891 Sig-ma-Aldrich) and methylumbelliferone (MUF) (M1381 Sigma-Aldrich) were used to normalize the emitted fluorescence by the potential activities mentioned. Both, substrates analogues and standards were dissolved in 2-methoxyethanol. Briefly, in sterile 96 well microplates with F bottom and low protein binding (XT64.1, Carl Roth), 300 μL of only liquid culture was added as blank. Moreover, 15 μL of the respective standard was added to 285 μL of liquid culture. For MCA, the final concentrations were 100 μM, 50 μM, 10 μM, and 1 μM, and for MUF, 2000 μM, 1000 μM, 100 μM, and 50 μM. Finally, 30 μL of the respective fluorogenic substrate was added to 270 μL of liquid culture and serially diluted to obtain 12 final concentrations from 1000 to 0.5 μM, except trypsin, from 500 to 0.2 μM. The volume was completed with an additional 150 μL of liquid culture. All these enzymatic assays were done with biological triplicates, The emitted fluorescence was measured hourly with FluoroLog^®^ Horiba at an excitation wavelength of 365 nm and an emission wavelength of 445 nm for 3 hours (T_0_, T_1_, T_2_, and T_3_). Between measurements, the microplates were incubated in the dark at 5°C.

**Table 1:** Enzymes targeted with fluorogenic substrate analogues of three categories, and their respective fluorogenic standards (MUF methylumbelliferyl, and MCA methylcoumarylamide).

Category	ID	Enzyme	Standard
Carbohydrates	BGL	β-glucosidase	MUF
	BXY	β-xylosidase	MUF
	NAG	*N*-acetyl-β-D-glucosaminidase	MUF
Lipids, phosphorus and sulfur moieties	OLE	Lipase	MUF
	APA	Alkaline phosphatase	MUF
	SUL	Sulfatase	MUF
Proteins	LAP	Leucine aminopeptidase	MCA
	TRY	Trypsin	MCA

The abbreviations of the respective enzyme used throughout the text are given in the column ID.

To calculate the kinetic parameters such as maximum velocity (V_max_) and half-saturation constant (K_m_), the hydrolysis rates obtained from the change of fluorescence over time in the samples with the fluorogenic substrate, were fitted directly to the Michaelis–Menten equation using nonlinear least-squares regression analysis in RStudio (Huitema and Horsman, 2018). For the biomass-specific activity, the V_max_ was normalized by the dry weight [μmol/g biomass*h], and for the cell-specific activity, it was normalized by the cell abundance [amol/cell*h]. The V_max_ and K_m_ values provided in this study refer only to the cell-free fraction, as the total EEA values are from Salazar-Alekseyeva et al. (2023) [in revision]. The percentage of the cell-free fraction to the total EEA (cell-attached plus cell-free) was calculated by comparing the cell-free rates from this study to the total rates from the mentioned study of Salazar-Alekseyeva et al. (2023) [in revision], all of them normalized by the biomass. To evaluate the distribution of the obtained data, Shapiro-Wilk test was used. Additionally, the significance between fungal species of these kinetic parameters and percentages was analyzed with one-way Analysis of Variance (ANOVA). Finally, Tukey’s Honestly Significant Difference (Tukey’s HSD) and Student-T were performed to identify significance at species level. All statistical analysis were ran in RStudio.

## 3 Results

**Figure 1:**
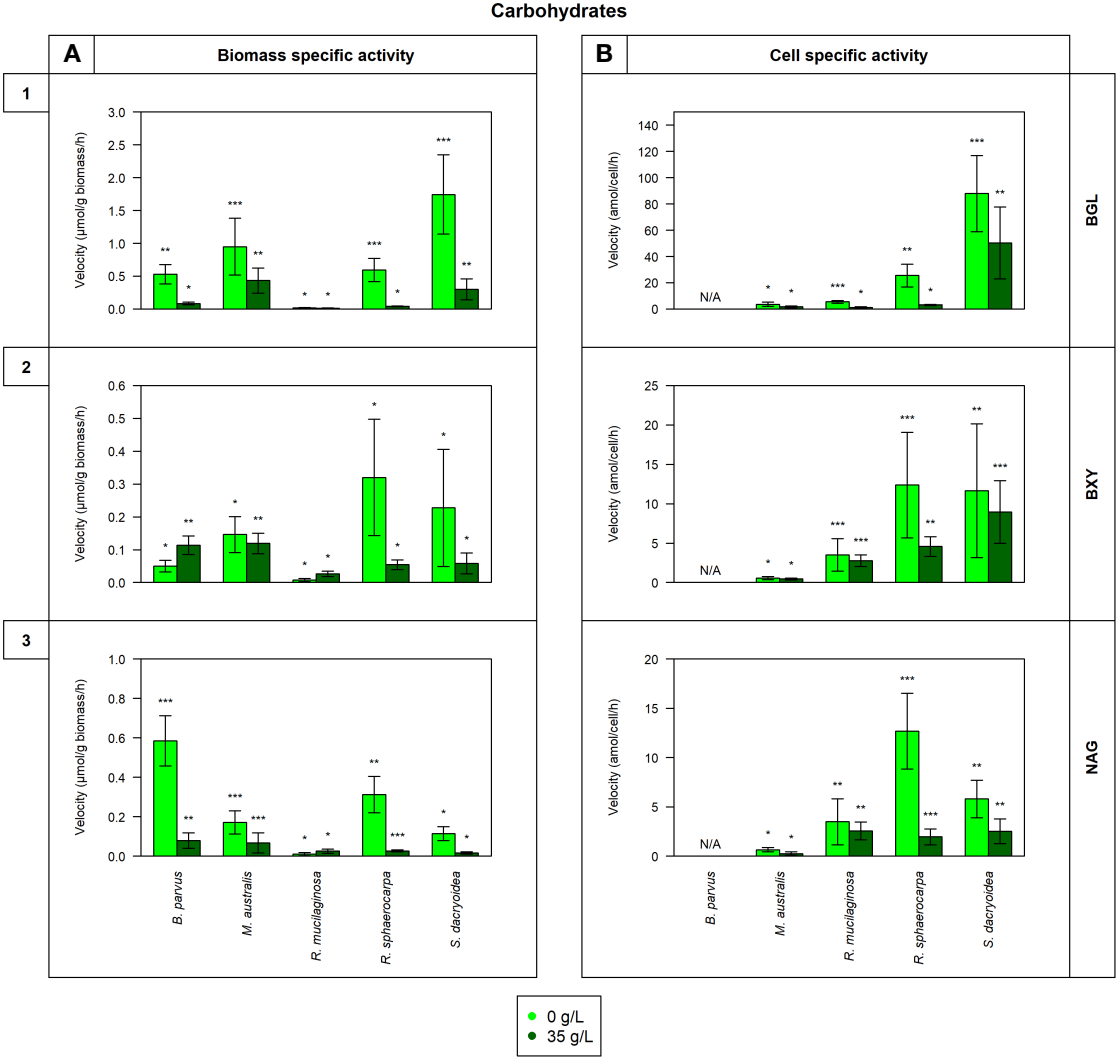
Maximum velocity (V_max_) of the cell-free enzymatic activity obtained from the filtrate of biological triplicates of the fungal strains *B. parvus*, *M. australis*, *R. mucilaginosa*, *R. sphaerocarpa*, and *S. dacryoidea* grown under non-saline (light green) and saline (dark green) conditions, and normalized by the **(A)** dry weight in μmol/g biomass*h and by the **(B)** cell abundance in amol/cell*h. For *B. parvus*, **B** was not possible to calculate, so it is represented by “N/A”. The substrates hydrolyzed denoted the use of carbohydrates by (**1**) β-glucosidase (BGL), (**2**) β-xylosidase (BXY), and (**3**) *N*-acetyl-β-D-glucosaminidase (NAG). Moreover, Tukey’s HSD was calculated by salinity where * represents p < 0.05; ** p < 0.01; and *** p < 0.001.

**Figure 2:**
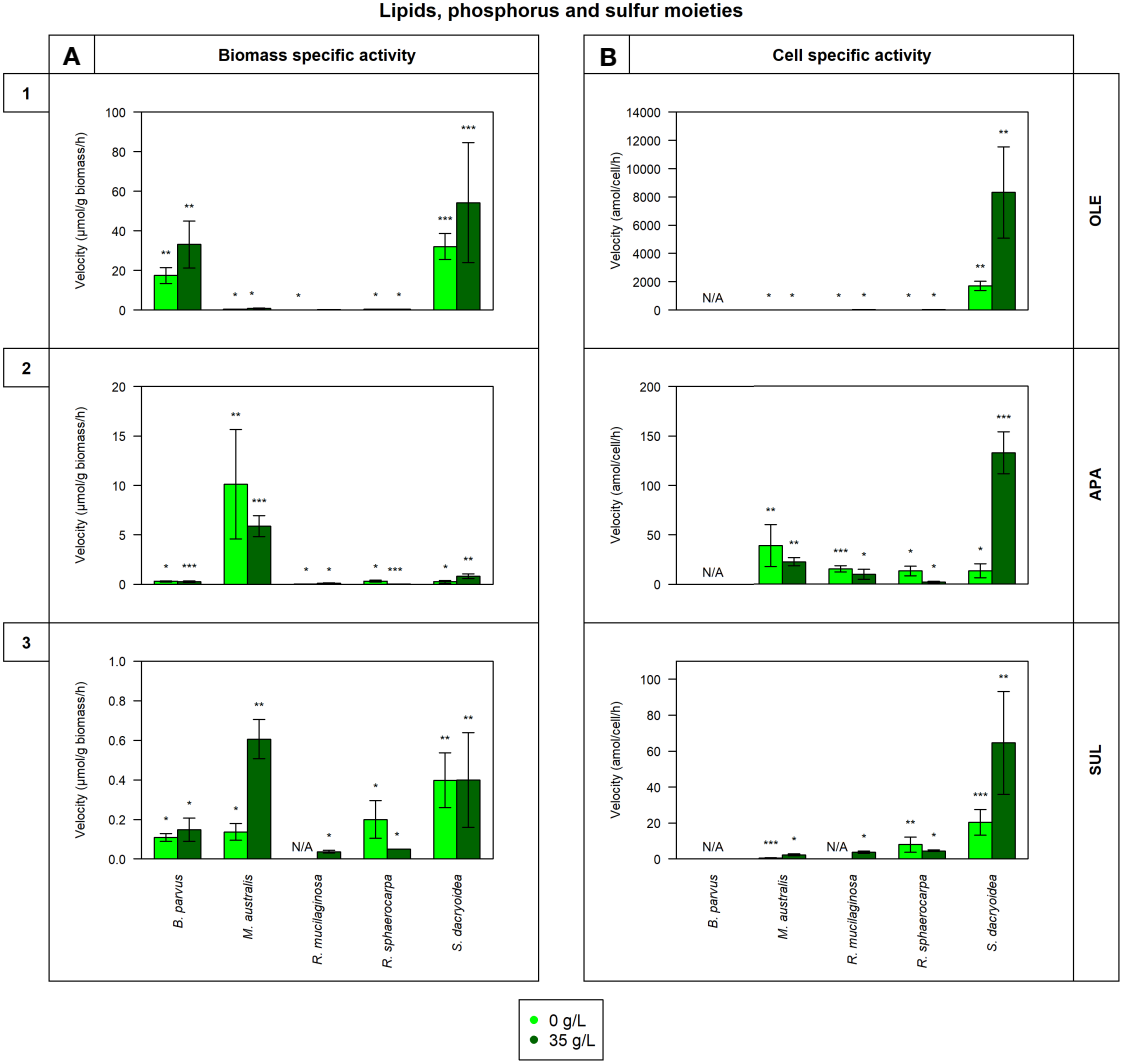
Maximum velocity (V_max_) of the cell-free enzymatic activity obtained from the filtrate of biological triplicates of the fungal strains *B. parvus*, *M. australis*, *R. mucilaginosa*, *R. sphaerocarpa*, and *S. dacryoidea* grown under non-saline (light green) and saline (dark green) conditions, and normalized by the **(A)** dry weight in μmol/g biomass*h and by the **(B)** cell abundance in amol/cell*h. For *B. parvus*, **B** was not possible to calculate, so it is represented by “N/A”. The substrates hydrolyzed denoted the use of lipids, phosphorus and sulfur moieties by (**1**) lipase (OLE), (**2**) alkaline phosphatase (APA), and (**3**) sulfatase (SUL), respectively. *R. mucilaginosa* did not exhibit any SUL activity under non-saline conditions, so it is also represented by “N/A”. Moreover, Tukey’s HSD was calculated by salinity where * represents p < 0.05; ** p < 0.01; and *** p < 0.001.

**Figure 3:**
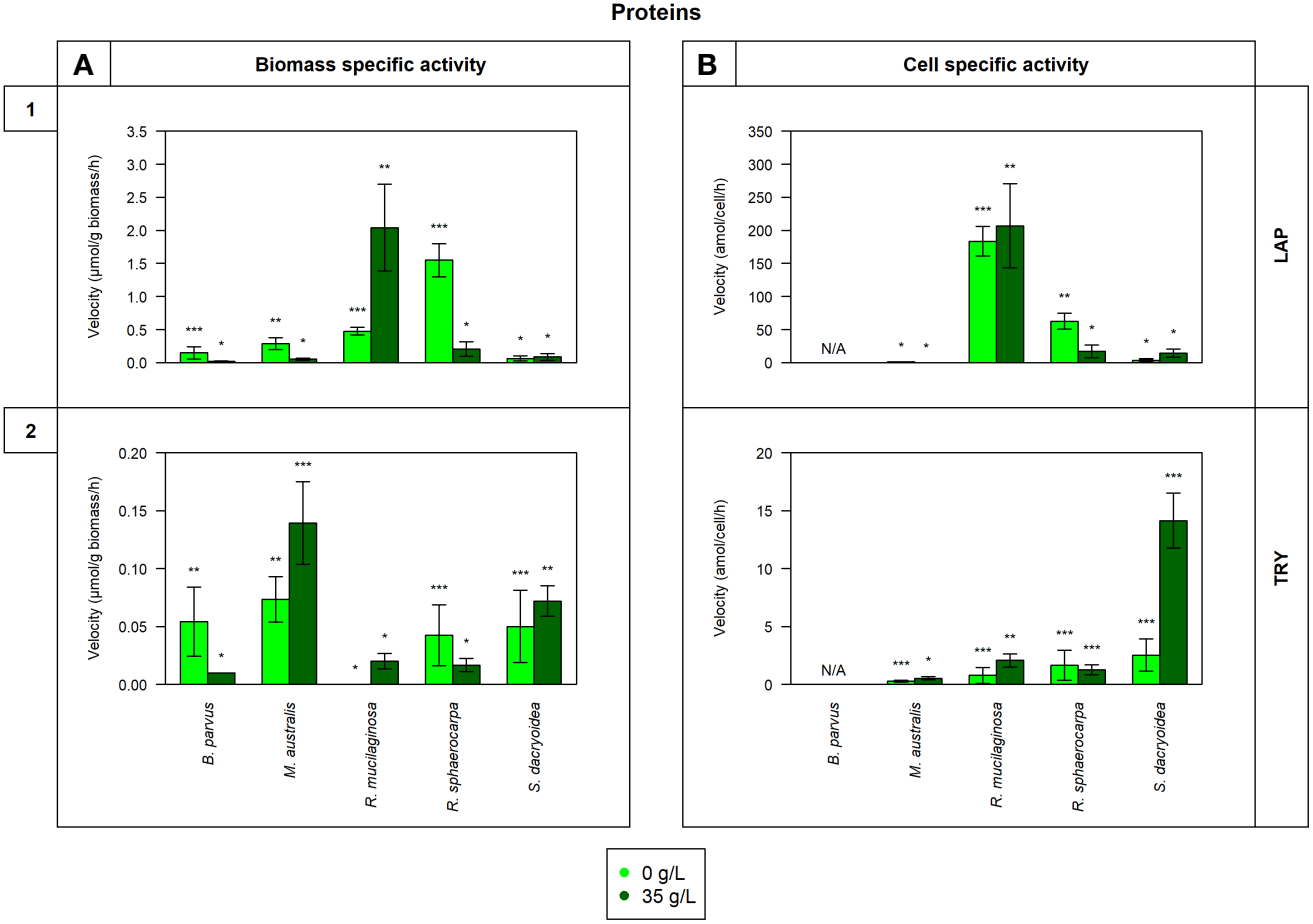
Maximum velocity (V_max_) of the cell-free enzymatic activity obtained from the filtrate of biological triplicates of the fungal strains *B. parvus*, *M. australis*, *R. mucilaginosa*, *R. sphaerocarpa*, and *S. dacryoidea* grown under non-saline (light green) and saline (dark green) conditions and normalized by the **(A)** dry weight in μmol/g biomass*h and by the **(B)** cell abundance in amol/cell*h. For *B. parvus*, **B** was not possible to calculate, so it is represented by “N/A”. The substrates hydrolyzed denoted the use of proteins by (**1**) leucine aminopeptidase (LAP), and (**2**) trypsin (TRY). Moreover, Tukey’s HSD was calculated by salinity where * represents p < 0.05; ** p < 0.01; and *** p < 0.001.

**Figure 4:**
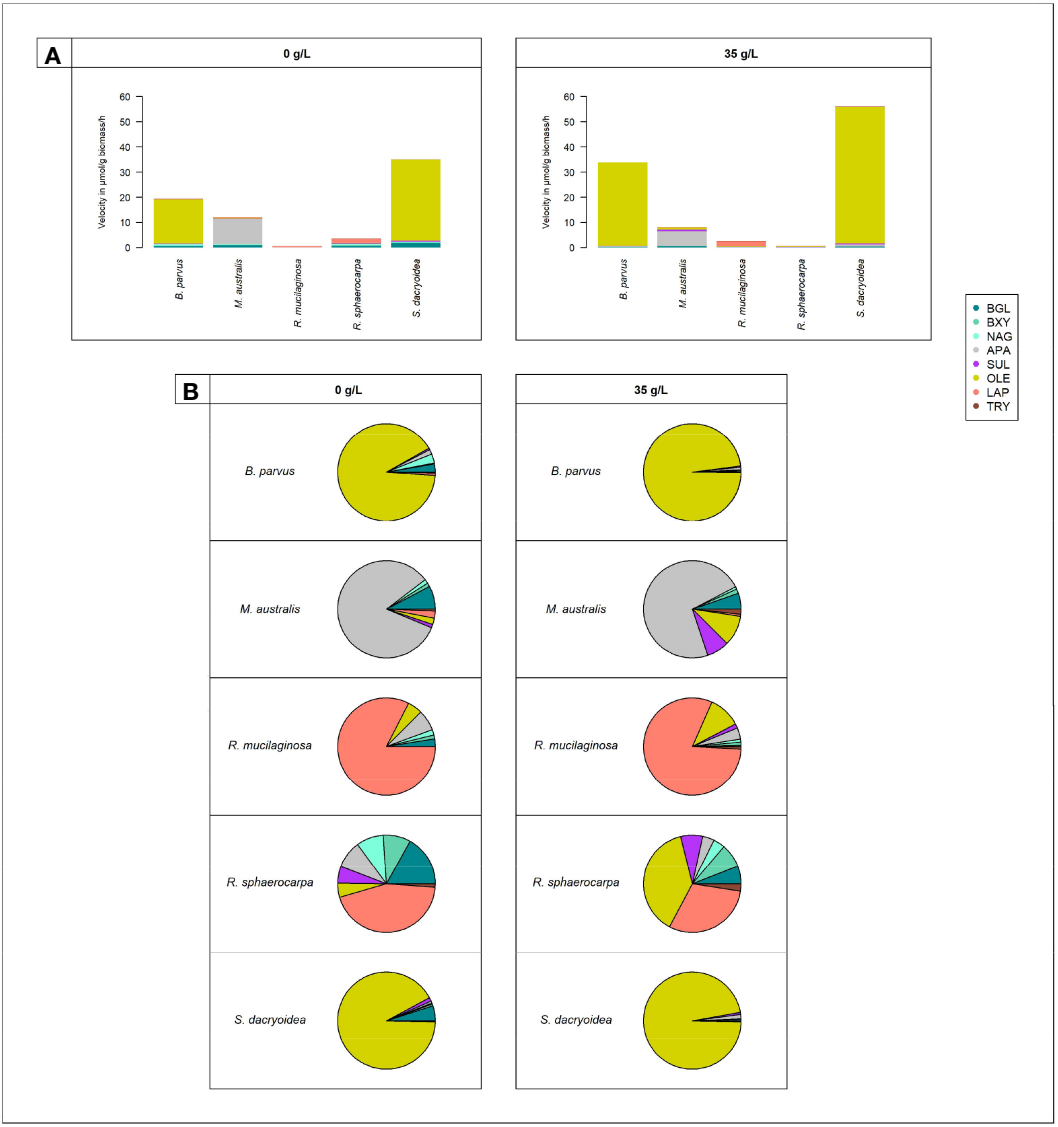
Cell-free enzymatic activities by fungal species: *B. parvus*, *M. australis*, *R. mucilaginosa*, *R. sphaerocarpa*, and *S. dacryoidea* for β-glucosidase (BGL), β-xylosidase (BXY), *N*-acetyl-β-D-glucosaminidase (NAG), lipase (OLE), alkaline phosphatase (APA), sulfatase (SUL), leucine aminopeptidase (LAP), and trypsin (TRY), under non-saline (0 g/L), and saline (35 g/L) conditions. **(A)** Sum of all cell-free V_max_ in μmol/g biomass*h and **(B)** Percentage of all cell-free V_max_ in %.

**Figure 5:**
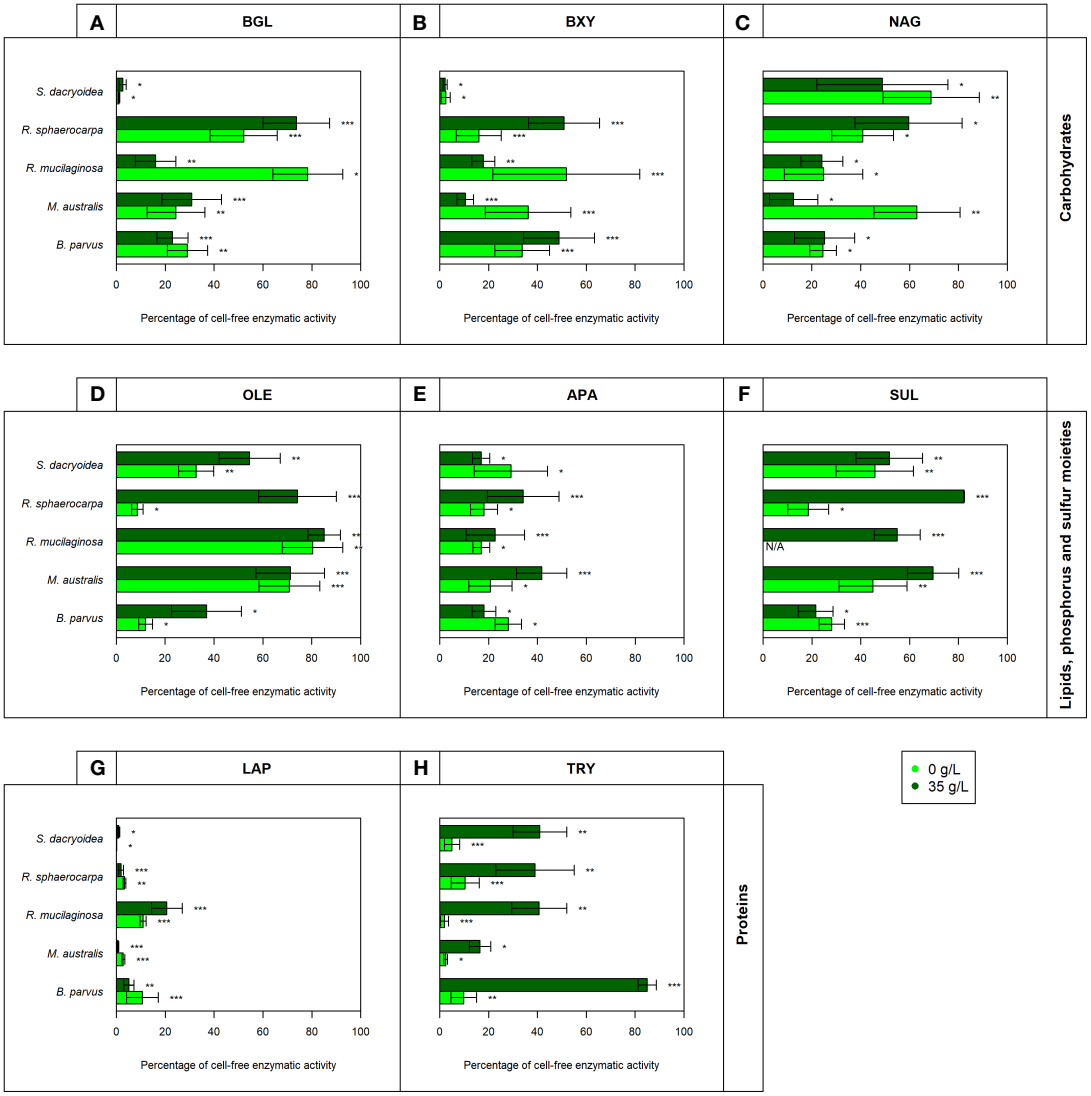
Contribution of cell-free enzymatic activity as a percentage to the total extracellular enzymatic activity normalized by the biomass for the enzymes **(A)** β-glucosidase (BGL), **(B)** β-xylosidase (BXY), **(C)** *N*-acetyl-β-D-glucosaminidase (NAG), **(D)** lipase (OLE), **(E)** alkaline phosphatase (APA), **(F)** sulfatase (SUL), **(G)** leucine aminopeptidase (LAP), and **(H)** trypsin (TRY) of biological triplicates of the fungal strains *B. parvus*, *M. australis*, *R. mucilaginosa*, *R. sphaerocarpa*, and *S. dacryoidea*. The species *R. mucilaginosa* did not exhibit any SUL activity under non-saline conditions, so it is represented by “N/A”. According to the salinity, non-saline (light green) and saline (dark green), Tukey’s HSD was performed and * represents p < 0.05; ** p < 0.01; and *** p < 0.001.

Remarkably, all the studied fungal species were capable to release cell-free enzymes to enzymatically hydrolyze carbohydrates (**Figure 1**), lipids, phosphorus and sulfur moieties (**Figure 2**), and proteins (**Figure 3**). Generally, the contribution of cell-free enzymes to the total EEA (V_max_) varied among the fungal species, as well as between the non-saline and saline conditions (**Figure 4** and **Table 2**), similar to the K_m_ values (**Table 3**). The percentage of cell-free enzyme secretion represented up to 85.1% of the total EEA (**Figure 5**).

**Table 2:** Main location (≥50%), cell-attached (CA) or cell-free (CF), of the enzymes degrading carbohydrates by β-glucosidase (**BGL**), β-xylosidase (**BXY**), and *N*-acetyl-β-D-glucosaminidase (**NAG**); lipids, phosphorus and sulfur moieties by lipase (**OLE**), alkaline phosphatase (**APA**), and sulfatase (**SUL**), respectively; and proteins by leucine aminopeptidase (**LAP**) and trypsin (**TRY**).

	Carbohydrates						Lipids, phosphorus and sulfur moieties						Proteins			
**Enzyme**	**BGL**		**BXY**		**NAG**		**APA**		**SUL**		**OLE**		**LAP**		**TRY**	
**Condition**	**N**	**S**	**N**	**S**	**N**	**S**	**N**	**S**	**N**	**S**	**N**	**S**	**N**	**S**	**N**	**S**
*B. parvus*	CA	CA	CA	CA	CA	CA	CA	CA	CA	CA	CA	CA	CA	CA	CA	CF
*M. australis*	CA	CA	CA	CA	CF	CA	CA	CA	CA	CF	CF	CF	CA	CA	CA	CA
*R. mucilaginosa*	CF	CA	CF	CA	CA	CA	CA	CA	N/ A	CF	CF	CF	CA	CA	CA	CA
*R. sphaerocarpa*	CF	CF	CA	CF	CA	CF	CA	CA	CA	CF	CA	CF	CA	CA	CA	CA
*S. dacryoidea*	CA	CA	CA	CA	CF	CA	CA	CA	CA	CA	CA	CF	CA	CA	CA	CA

These were investigated in five marine fungal isolates *B. parvus*, *M. australis*, *R. mucilaginosa*, *R. sphaerocarpa*, and *S. dacryoidea* grown under non-saline (**N**) and saline (**S**) conditions. *R. mucilaginosa* did not exhibit any SUL activity under non-saline conditions, so it is indicated below by “N/A”.

**Table 3:** Average and standard deviation of K_m_ in µM calculated from the cell-free enzymatic activity fraction of biological triplicates of five marine fungal isolates *B. parvus*, *M. australis*, *R. mucilaginosa*, *R. sphaerocarpa*, and *S. dacryoidea* grown under non-saline (**N**) and saline (**S**) conditions.

Species		*B. parvus*		*M. australis*		*R. mucilaginosa*		*R. sphaerocarpa*		*S. dacryoidea*	
Condition		N	S	N	S	N	S	N	S	N	S
**Carbohydrates**	**BGL**	288.7 ± 111.1	472.6 ± 171.1	466.8 ± 59.3	366.7 ± 98.7	363.3 ± 215.8	8.3 ± 4.1	26.6 ± 4.8	12.1 ± 5.3	635.9 ± 81.3	398.1 ± 81.5
	**BXY**	40.2 ± 24.0	13.7 ± 8.7	10.5 ± 3.3	6.7 ± 2.6	11.4 ± 2.9	16.7 ± 11.2	6.7 ± 1.6	8.7 ± 3.7	16.2 ± 6.4	25.0 ± 14.8
	**NAG**	100.9 ± 27.1	648.6 ± 120.5	39.4 ± 7.8	27.4 ± 7.4	214.0 ± 57.9	294.0 ± 127.9	0.8 ± 0.2	6.6 ± 1.8	39.8 ± 18.9	13.8 ± 4.4
**Lipids, phosphorus and sulfur moieties**	**APA**	24.4 ± 8.6	300.9 ± 161.4	117.8 ± 37.8	47.5 ± 7.8	379.3 ± 173.9	115.9 ± 64.0	27.7 ± 13.3	1.3 ± 0.4	4.3 ± 2.0	9.2 ± 3.7
	**SUL**	32.4 ± 15.2	384.7 ± 146.8	121.5 ± 65.1	413.7 ± 200.8	N/A	566.0 ± 189.3	1.1 ± 0.3	1.5 ± 0.6	345.4 ± 135.5	572.9 ± 171.8
	**OLE**	60.9 ± 16.1	59.8 ± 21.6	404.1 ± 203.3	161.7 ± 63.1	430.4 ± 165.6	602.2 ± 222.8	3.3 ± 1.4	146.1 ± 32.7	15.8 ± 4.2	61.2 ± 18.9
**Proteins**	**LAP**	316.1 ± 154.8	66.2 ± 24.8	48.0 ± 14.4	109.4 ± 3.3	74.1 ± 12.6	108.0 ± 44.2	133.2 ± 19.0	109.8 ± 26.2	347.5 ± 146.3	462.7 ± 140.1
	**TRY**	136.1 ± 79.8	3.1 ± 1.9	10.7 ± 5.0	58.3 ± 6.4	12.2 ± 2.9	13.2 ± 7.3	15.6 ± 1.9	3.2 ± 0.4	333.6 ± 119.7	83.1 ± 43.9

The substrates used were indicative of cleavage of carbohydrates by β-glucosidase (**BGL**), β-xylosidase (**BXY**), and *N*-acetyl-β-D-glucosaminidase (**NAG**); lipids, phosphorus and sulfur moieties by lipase (**OLE**), alkaline phosphatase (**APA**), and sulfatase (**SUL**), respectively; and proteins by leucine aminopeptidase (**LAP**), and trypsin (**TRY**). *R. mucilaginosa* did not exhibit any SUL activity under non-saline conditions, so it is indicated below by “N/A”.

### 3.1 Cleavage of carbohydrates

#### 3.1.1 β-glucosidase

In all the fungal species studied, the V_max_ of cell-free BGL was significantly higher in the non-saline than in the saline medium (*t-test*; p < 0.001; **Figure 1.1**), but there was no significant difference in the K_m_ (*t-test*; p= 0.3; **Table 3**). The highest V_max_ value was detected in *S. dacryoidea* under non-saline conditions (*t-test*; p < 0.001; 1.7 ± 0.6 μmol/g biomass*h and 87.9 ± 29.0 amol/cell*h). The percentage of cell-free relative to the total BGL by *S. dacryoidea* only represented 1.0 ± 0.4% and 2.6 ± 1.5% in the non-saline and saline medium, respectively (**Figure 5A**). In contrast, in the two species of the genus *Rhodotorula*, *R. mucilaginosa* under non-saline conditions and *R. sphaerocarpa* under saline conditions, the BGL rates were low, but the proportion of cell-free fraction represented 78.3 ± 14.3%, and 73.6 ± 13.7%, respectively, of the total BGL. For the remaining species, the contribution of cell-free to the total BGL activities ranged between 16.0% and 52.1%.

#### 3.1.2 β-xylosidase

Similar to BGL, in all the fungal strains, the V_max_ of cell-free BXY was significantly higher under non-saline than under saline conditions (*t-test*; p= 0.005; **Figure 1.2**), but no significant difference was detected in the K_m_ (*t-test*; p= 0.5; **Table 3**). In the non-saline medium, the highest V_max_ values were detected for *R. sphaerocarpa* and *S. dacryoidea* (*t-test*; p= 0.001), with values of 0.3 ± 0.2 μmol/g biomass*h and 12.4 ± 6.7 amol/cell*h, and 0.2 ± 0.2 μmol/g biomass*h and 11.7 ± 8.5 amol/cell*h, respectively. Consistent with what was observed for BGL, even though *S. dacryoidea* exhibited one of the highest V_max_, the contribution of the cell-free fraction to the total EEA was low with percentages of 2.4 ± 1.9% and 2.2 ± 0.8% under non-saline and saline conditions, respectively (**Figure 5B**). For the remaining fungal strains, the contribution of cell-free to the total BXY varied between 10.4 and 51.8%.

#### 3.1.3 *N*-acetyl-β-D-glucosaminidase

Similar to BGL and BXY, the V_max_ of cell-free NAG was significantly higher in the non-saline than in the saline medium for all the species studied (*t-test*; p < 0.001; **Figure 1.3**). However, as shown in **Table 3**, there was no significant difference in the K_m_ for all the species (*t-test*; p= 0.3), except for *B. parvus*, where it was significantly higher under saline conditions (*t-test*; p= 0.02; 631.5 ± 141.5 μM). When the V_max_ was normalized by the biomass, *B. parvus* had the highest V_max_ under non-saline conditions (*t-test*; p < 0.001; 0.6 ± 0.1 μmol/g biomass*h). But when the V_max_ was normalized by the cell abundance, *R. sphaerocarpa* exhibited the highest V_max_ also in this medium (*t*-test; p= 0.003; 12.7 ± 3.8 amol/cell*h). The contribution of the cell-free fraction to the total NAG varied from 12.5 to 68.8% (**Figure 5C**).

### 3.2 Cleavage of lipids, phosphorus and sulfur moieties

#### 3.2.1 Lipase

The V_max_ values were significantly higher in the saline than in the non-saline medium for all the fungal strains (*t-test*; p= 0.05; **Figure 2.1**). The K_m_, however, was not significantly different between both media (*t-test*; p= 0.4; **Table 3**). *S. dacryoidea* exhibited significantly higher V_max_ than the other species (*t-test*; p < 0.001), with values of 32.1 ± 6.5 μmol/g biomass*h and 1676.5 ± 333.4 amol/cell*h under non-saline conditions, and 54.2 ± 30.3 μmol/g biomass*h and 8319.8 ± 3229.3 amol/cell*h under saline conditions. The contribution of cell-free to the total OLE oscillated from 8.6 to 85.1% where the highest percentages corresponded to species with generally low OLE activity, such as *M. australis, R. mucilaginosa*, and *R. sphaerocarpa* (**Figure 5D**).

#### 3.2.2 Alkaline phosphatase

The V_max_ of cell-free APA was significantly higher under non-saline than under saline conditions for all the fungal strains (*t-test*; p< 0.001; **Figure 2.2**), but no significant difference was detected in the K_m_ (*t-test*; p= 0.6; **Table 3**). Normalizing APA activity to the biomass revealed a significantly higher V_max_ in *M. australis* under both, non-saline and saline conditions (*t-test*; p < 0.001; 10.1 ± 5.5 μmol/g biomass*h and 5.9 ± 1.1 μmol/g biomass*h, respectively). Normalizing the V_max_ to the cell abundance exposed *S. dacryoidea* in the saline medium as the species with the highest V_max_ (*t-test*; p < 0.001; 132.5 ± 21.1 amol/cell*h). The contribution of cell-free to the total APA varied between 16.9 and 41.7% (**Figure 5E**).

#### 3.2.3 Sulfatase

All the marine fungal strains expressed cell-free SUL activity with no significant difference between the V_max_ in the non-saline and saline medium (*t-test*; p= 0.5; **Figure 2.3**). The highest SUL activity detected among all the fungal strains was in *S. dacryoidea* under saline conditions (*t-test*; p= 0.04; 0.4 ± 0.2 μmol/g biomass*h and 64.6 ± 28.6 amol/cell*h). Contrary, in the case of the K_m_, these values were significantly higher under saline than under non-saline conditions for all the species (*t-test*; p< 0.001; **Table 3**). The proportion of the cell-free fraction of SUL to the total SUL activity varied between 18.5 and 82.3% (**Figure 5F**).

### 3.3 Cleavage of proteins and peptides

#### 3.3.1 Leucine aminopeptidase

There was no significant difference in the V_max_ nor the K_m_ of cell-free LAP between the non-saline and saline medium for none of the fungal species (*t-test*; p= 0.4; **Figure 3.1** and *t-test*; p= 0.9; **Table 3**, respectively). The highest LAP activity detected among all the fungal strains was in *R. mucilaginosa* under saline conditions (*t-test*; p < 0.001; 2.0 ± 0.7 μmol/g biomass*h and 206.8 ± 63.6 amol/cell*h). The highest contribution of cell-free LAP to the total LAP activity was also detected in *R. mucilaginosa* (20.6 ± 6.3%). The other fungal species exhibited generally a low contribution of cell-free LAP activity to the total LAP activity ranging from 0.1 to 10.9% (**Figure 5G**).

#### 3.3.2 Trypsin

There was no significant difference between the V_max_ obtained in the non-saline and the saline medium (*t-test*; p= 0.08; **Figure 3.2**). However, as shown in **Table 3**, *M. australis*, *R. mucilaginosa*, and *R. sphaerocarpa* exhibited a significantly higher K_m_ in the saline than in the non-saline medium (*t-test*; p= 0.04). The contrary occurred for *B. parvus* and *S. dacryoidea* (*t-test*; p= 0.003). Similar to APA, TRY activity normalized by the biomass resulted in a significantly higher V_max_ for *M. australis* in both media than for the remaining species (*t-test*; p< 0.001). However, normalizing the V_max_ to cell abundance revealed that *S. dacryoidea* in the saline medium exhibited a higher V_max_ than the other fungal species (*t-test*; p < 0.001; 14.1 ± 2.4 amol/cell*h). Interestingly, TRY was the only enzyme studied where a significant difference in the contribution of cell-free TRY activity to the total TRY activity was detectable between the non-saline and saline medium (**Figure 5H**). The contribution of the cell-free fraction to the total TRY activity was significantly higher under saline with 16.5 to 84.9% than under non-saline conditions with 2.5 to 10.4% (*t-test*; p < 0.001).

### 3.4 Cleavage of proteins and peptides

Compared to the other EEAs tested, the contribution of cell-free OLE to the cell-free enzymatic pool was significantly higher (*t-test*; p < 0.001; **Figure 4**). Cell-free OLE exhibited the highest V_max_ values, and these were mainly found in *S. dacryoidea* and *B. parvus*. Contrarily, the contribution of cell-free TRY to the cell-free enzymatic pool was significantly lower than the other EEAs (*t-test*; p < 0.001; **Figure 4**).

## 4 Discussion

### 4.1 Release of cell-free enzymes by pelagic fungal strains

It is remarkable that all the species studied of marine pelagic fungi, *B. parvus*, *M. australis*, *R. mucilaginosa*, *R. sphaerocarpa*, and *S. dacryoidea*, released cell-free enzymes. These species seem to be versatile as they exhibited a spectrum of cell-free enzymes capable of hydrolytically cleaving carbohydrates, proteins, lipids, and moieties of phosphorus and sulfur at a distance from the cell that originally produced them. Nonetheless, species and enzymes were affected differently by salinity changes.

#### 4.1.1 Release of cell-free enzymes cleaving carbohydrates

Cellulose is a polymeric substrate that cannot be transported across the fungal cell wall, requiring at least partial extracellular hydrolysis (MacDonald and Speedie, 1982). The release of cell-free β-glucosidase by fungi has been reported in terrestrial species like *Sporotrichum thermophile* (Bhat et al., 1993; Gaikwad and Maheshwari, 1994) and marine species associated with decaying estuarine and marine plants (MacDonald and Speedie, 1982), driftwood (MacDonald and Speedie, 1982), mangroves (Pointing et al., 1999), sediments (Elyas et al., 2010), and macroalgae (Lee et al., 2019). In the present study, the level of secretion of cell-free β-glucosidase differed among the species studied (**Table 2**). Compared with the rest of the fungal species, *S. dacryoidea* was the species that exhibited the highest V_max_ of cell-free (**Figure 1.1**), but the lowest cell-free contribution to the total BGL (**Figure 5A**). This low cell-free contribution to the total β-glucosidase activity has also been reported for species like *Trichocladium achrasporum* (MacDonald et al., 1985). For the other fungal species tested except the ones of the genus *Rhodotorula*, *R. mucilaginosa* and *R. sphaerocarpa*, the cell-free fraction represented up to 30.8% of the total EEA suggesting that the β-glucosidase activity is mainly cell-associated (**Table 2** and **Figure 5A**). This is consistent with previous reports on other marine fungi species (MacDonald and Speedie, 1982; MacDonald et al., 1985; Pointing et al., 1999).

Like cellulose, xylan also cannot penetrate the cell due to its polymeric structure (Biely, 1985; Lenartovicz et al., 2003; Collins et al., 2005), so β-xylosidases can be cell-attached or cell-free (Lenartovicz et al., 2003). Reese et al. (1973) reported that in the early growth stages of fungal cultures, xylosidases were cell-attached, but later on, these enzymes were released into the medium either by true secretion or cell lysis. Interestingly, similar to BGL, although *S. dacryoidea* displayed one of the highest V_max_ values (**Figure 1.2**), cell-free β-xylosidase was low compared to the total EEA (**Figure 5B**). Hence, we conclude that β-xylosidases released by the studied marine fungi species were also mostly cell-attached (**Table 2**). This was also found for some widespread fungi species like *Cryptococcus albidus* (Defaye et al., 1992), *Aspergillus fumigatus* (Lenartovicz et al., 2003), *Thermomyces lanuginosus* (Singh et al., 2003), *Aureobasidium pullulans* (Ohta et al., 2010), and some other species (Reese et al., 1973).

In the study of Matsumoto et al. (2004), *Verticillium lecanii*, originally isolated from *Lecanium corni*, produced extracellular *N*-acetyl-β-D-glucosaminidase from shrimp waste. Fungi can degrade chitin and use it as a carbon and nitrogen source (Gaderer et al., 2017), but in contrast to bacteria, fungi can also use it as a building block for the synthesis of new chitin (Edson and Brody, 1976). Thus, the number of chitinases produced by fungi has been related to their chitin content and growth mode (Hartl et al., 2012). For instance, as the cell wall of filamentous fungi consists of 10 to 20% of chitin (Ruiz-Herrera, 1991), the number of chitinases is normally 10 to 30 (Seidl, 2008; Kubicek et al., 2011). The cell wall of yeast fungi consists of only 0.5 to 5% of chitin (Garcia-Rubio et al., 2020), so the number of chitinases might be lower in yeast than in filamentous fungi. Even though the overall *N*-acetyl-β-D-glucosaminidase activity was low, compared with other species, *B. parvus*, a filamentous species, exhibited the highest V_max_ of cell-free (**Figure 1.3**) similar to what was reported by Salazar-Alekseyeva et al. (2022) and Salazar-Alekseyeva et al. (2023) [in revision]. As shown in **Table 2**, the main location of *N*-acetyl-β-D-glucosaminidase might be cell-attached.

Marine microorganisms require diverse substrates which are normally polymeric (Wang et al., 2016). In these environments, carbohydrates are the largest macromolecular compound class of DOC (Benner et al., 1992). According to Biely (1985), microorganisms that compete for carbon sources, secrete enzymes that are mainly cell-attached. As mentioned above, cellulose, chitin, and xylan are polymeric structures, so for these carbohydrates, fungal cell-attached enzymes might dominate the hydrolytic cleavage of this abundant macromolecular compound class.

### 4.1.2 Release of cell-free enzymes cleaving lipids, phosphorus and sulfur moieties

Lipids are high energy sources (Parrish, 2013), and also building blocks for organisms (Bergé and Barnathan, 2005), so the degradation of lipids like phospholipids might be a means to obtain both, carbon and phosphorus (Celussi and Del Negro, 2012). According to Singh and Mukhopadhyay (2012) and Duarte et al. (2021), the majority of lipases in fungi were released into the extracellular medium. In the studies of Papanikolaou et al. (2007) and Louhasakul et al. (2016), the marine yeast *Yarrowia lipolytica*, produced only cell-attached lipase, but in the study of Scioli and Vollaro (1997) this same species produced both, cell-attached and cell-free OLE. In another study, other 9 marine yeast strains, including one used in our study, *R. mucilaginosa*, produced cell-attached lipase, and only *Aureobasidium pullulans* produced cell-free lipase (Wang et al., 2007). **Table 2** and **Figure 5D** indicate that the lipase activity of *M. australis* and *R. mucilaginosa* was mainly cell-free, whereas for the species *R. sphaerocarpa*, and *S. dacryoidea* the cell-free fraction depended mainly on the salinity, where higher percentages of cell-free enzymatic activity were found under saline conditions. *B. parvus* was the only species where the majority of lipase activity was cell-attached. Hence, we suggest that the release of cell-free lipase might be species-specific and dependent on the salinity.

Phosphorus is an essential element required for many biological processes (Colman et al., 2005; Paytan and McLaughlin, 2007; Brembu et al., 2017; Lockwood et al., 2022). In aquatic environments, low phosphate concentrations have been related to the expression of alkaline phosphatase (Hassan and Pratt, 1977; Colman et al., 2005; Srivastava et al., 2021). However, in carbon-limited environments, microorganisms might use APA not only to obtain phosphate, but also to access the carbon moieties from organic matter (Hoppe and Ullrich, 1999; Colman et al., 2005). In bacteria, APA is generally located in the periplasmatic space, whereas in fungi, it is generally attached to the cell surface (Chrost et al., 1984). In soil studies, six fungal species, one of them a widespread fungus as it is *Aspergillus niger*, released approximately 22% of extracellular alkaline phosphatase (Tarafdar et al., 2002). These authors suggested that this low release was related to the fungal structure, and a low membrane permeability for this enzyme (Tarafdar et al., 2002). Our study on marine fungi also suggests that APA activity is mostly cell-attached (**Table 2**), as the activities of the cell-free APA fraction amounted to at most 41.7% of the total APA activity (**Figure 5E**).

Sulfur is also an essential element required for numerous biological molecules (Klotz et al., 2011; Helbert, 2017). In marine environments, polysaccharides can be highly sulfated (Kloareg and Quatrano, 1988; Wang et al., 2016; Helbert, 2017), probably as a physiological adaptation to the high environmental ionic strength (Kloareg and Quatrano, 1988; Aquino et al., 2005; Aquino et al., 2011; Ciancia et al., 2020). In these environments, the cleavage of sulfate groups might be necessary not only to obtain sulfur, but also to access the carbohydrates (Schultz-Johansen et al., 2018; Hettle et al., 2022). For the widespread fungi species *Neurospora crassa*, cell-attached, and cell-free sulfatase were reported by Scott and Metzenberg (1970). We also found these two types of extracellular enzymes in our studied marine fungi species. Interestingly, all the species, except *B. parvus*, released a higher percentage of cell-free enzymes in the saline than in the non-saline medium (**Table 2** and **Figure 5F**). Therefore, fungi might be capable to secrete cell-free sulfatase as a strategy to remove sulfates and gain access to other carbohydrates (Salazar-Alekseyeva et al., 2023) [in revision]).

#### 4.1.3 Release of cell-free enzymes cleaving proteins and peptides

Grazing and viral lysis are the main sources of proteins and peptides released into the seawater (Repeta, 2015). Proteases hydrolytically cleave them to obtain both, carbon and nitrogen (Li et al., 2019), and this can take place inside or outside the cell (Pantoja et al., 1997; Gupta et al., 2002). Nitrogen is an essential element, especially for the growth and function of enzyme-dependent microorganisms (Allison, 2005). Hydrolysis rates are mainly influenced by the size and chemical structure of the substrate, where peptides are hydrolyzed much faster than proteins (Pantoja and Lee, 1999). Bacterial leucine aminopeptidase has been widely reported to be mainly cell-attached in seawater (Pantoja et al., 1997; Obayashi and Suzuki, 2008b; Bong et al., 2013), as well as in freshwater (Chróst and Rai, 1993; Millar et al., 2015). Remarkably, in all the marine fungal strains studied, this was the only enzyme where the majority of enzymatic activity was cell-attached (up to 79.4%) in both non-saline and saline medium (**Table 2** and **Figure 5G**). *S. dacryoidea*, however, exhibited the lowest contribution of cell-free to the total LAP activity (**Figure 5G**). Trypsin also exhibited a high cell-attached enzymatic activity (up to 89.6%), but only under non-saline conditions (**Figure 5H**).

#### 4.1.4 Influence of salinity on cell-free enzymes in marine fungi

Diverse environmental factors can influence microbial cells, and also the subsequent secretion of enzymes (Chróst, 1990). In the case of cell-free enzymes, as these are released from the cell into the ambient water, their fate will depend on the conditions of the water (Hoppe and Ullrich, 1999; Baltar, 2018). Therefore, cell-free enzymes might be susceptible to degradation and chemical changes (MacDonald and Speedie, 1982; Wetzel, 1991).

Enzymes are strongly associated with water as it tends to bind to the hydrophobic groups located on the enzyme surface (Saenger, 1987; Kornblatt and Kornblatt, 2002; Zaccai, 2004; Rezaei et al., 2007). This allows the enzyme to maintain its native structure, and hence, it can function properly (Kuntz Jr, 1971; Saenger, 1987; Karan et al., 2012). As salts promote ionic and hydrophobic effects, salinity is considered an important environmental factor that can influence the solubility and stability of enzymes (Baxter, 1959; Lanyi, 1974; Karan et al., 2012) and thus, their functions (King, 1986; Caruso and Zaccone, 2000). Though, the magnitude of these effects will depend on the salt concentration as well as the chemical composition of the enzyme (Sinha and Khare, 2014). Some marine studies have reported salinity as an important environmental factor influencing microbial enzymatic activity (Caruso and Zaccone, 2000; Salazar-Alekseyeva et al., 2023)[in revision]). However, the influence of salinity on fungal cell-free enzymatic activities has not been reported yet. Based on our results, we suggest that the salinity effect on the kinetic parameters such as maximum velocity (V_max_) depends on the enzyme as well as on the species (**Figures 1**–**5**). Under saline conditions simulating marine environments, we found that the V_max_ of the enzymes BGL, BXY, NAG, and APA was reduced, with only one exception, the APA of *S. dacryoidea*. This reduction was different for each species, similar to what was reported for the total EEA by Salazar-Alekseyeva et al. (2023) [in revision].

Certain enzymes can be salt-tolerant or halophilic (Larsen, 1967). Here, amino acid residues located on the enzyme surface tend to bind to hydrated cations (Lanyi, 1974; Jin et al., 2019). This creates a large multilayered shell that keeps the enzyme hydrated (Karan et al., 2012), and also allows it to adopt a flexible conformational structure (Baxter, 1959; Hutcheon et al., 2005; Sinha and Khare, 2014; Karan et al., 2020). In the present study, under saline conditions, we detected potentially halophilic cell-free enzymes which were APA, SUL, and TRY for *S. dacryoidea*, SUL for *R. mucilaginosa*, and OLE for all the species (**Figures 2**, **3**). *S. dacryoidea* is a widespread species (Fell et al., 1973; Gadanho et al., 2003; Allen et al., 2004; Francis et al., 2016), that has been identified as a potentially facultative marine species adapted to varying salinities Salazar-Alekseyeva et al. (2023) [in revision]. As the available nutritional sources are found in high concentrations of osmolytes, fungi probably evolved enzymes to successfully compete for their uptake with other microorganisms (Gladfelter et al., 2019; Gonçalves et al., 2021). Curiously, cell-free lipase was the only enzyme where the V_max_ of all the studied fungal species was enhanced under saline conditions (**Figure 2.1**). Comparable results were supported by Kiran et al. (2009) showing that the production of lipase and other biosurfactants by the marine fungi *Aspergillus ustus* isolated from a symbiosis with a marine sponge, was higher under saline conditions. In the lipases of a pathogenic species, *Candida rugosa*, structural differences in the flap, substrate-binding pocket, and mouth of the hydrophobic tunnel, were responsible for three isoenzymes with different substrate specificity and catalytic properties (Mancheño et al., 2003). Moreover, the species *Yarrowia lipolytica* was reported to encode lipase isoenzymes with different substrate specificities (Fickers et al., 2005). On the other hand, interestingly, trypsin was the only enzyme that exhibited a higher percentage of cell-free activity under saline than under non-saline conditions (**Figure 5H**). In coastal seawater, cell-free trypsin has been reported to be between 40 to 80% of the total trypsin activity (Obayashi and Suzuki, 2008b). As a result, the release of cell-free enzymes into the surrounding water might be an advantage in marine environments allowing fungi to access substrates away from them.

In the open ocean, microbial cell-free enzymatic activities have been reported to be lower than in nearshore waters (Li et al., 2019). The reason for this might be the input of terrestrial substrates (Allison et al., 2012; Millar et al., 2015) and other organisms that stimulate the enzymatic activities in coastal waters (Li et al., 2019). For marine aggregates, Ziervogel and Arnosti (2008) stated that salinity or other chemical and physical factors can affect the lifetime of cell-free enzymes, as longer active lifetimes were found in nearshore waters than in offshore waters of the Gulf of Mexico. Our results indicate that salinity can influence the kinetics of cell-free enzymes, but marine fungi might be capable to produce different enzymes adapted to different salinities. For instance, in a study of fungi inhabiting mangrove forests, one fungal species was able to secrete two enzymes under saline conditions, and a different one in the absence of salt (Li et al., 2002). As proposed by Zaccai (2004), each enzyme might evolve a stable form specific to an environment, so isoenzyme expression might be a strategy to adapt to different salinities (Arfi et al., 2013; Salazar-Alekseyeva et al., 2023) [in revision]). From our study, it appears that the marine fungi species are adapted to a wide range of salinities, probably with different enzymes, also known as isoenzymes, capable to perform the same reaction under different salinities.

### 4.2 Location of marine fungi enzymes

The location of extracellular enzymes, either cell-attached or cell-free, influences the degradation and subsequent utilization of the substrates (Parawira et al., 2005), and this might depend on the lifestyle (Traving et al., 2015). For particle-associated microorganisms, cell-free enzymes could be favourable due to their proximity to the substrates (Vetter et al., 1998). Contrarily, for free-living microorganisms, cell-attached enzymes could be beneficial (Chróst, 1990; Traving et al., 2015) as the substrates in marine environments are generally highly diluted (McCarthy et al., 1996; Amon and Benner, 2003). For fungi, the location might be specially important (Dubovenko et al., 2010). In the case of marine fungi, a high secretion of CAZymes was associated with a preferential particle-associated lifestyle (Baltar et al., 2021). In the present study, we found that the majority of EEAs were cell-attached (**Table 2**), which suggests a free-living lifestyle. However, salinity might have influenced the switch to cell-free. For example, under non-saline conditions, the release of SUL and OLE was mainly cell-attached, but under saline conditions, the main location of these enzymes changed to cell-free. As mentioned before, highly sulfated polysaccharides are present in marine environments (Kloareg and Quatrano, 1988; Wang et al., 2016; Helbert, 2017), so a high salinity might induce a higher expression of cell-free enzymes like sulfatase. This also suggests that the fungal lifestyle might depend on the available substrates.

Unlike bacteria, fungi are capable to penetrate solid substrates (Raghukumar, 2017), specially the filamentous ones (Souza et al., 2015). In the present study, *B. parvus* was the only species that had this structure, and the majority of its EEAs were cell-attached (**Table 2**). These enzymes are capable of degrading substrates until they can be assimilated by the cells that originally synthesized them (MacDonald and Speedie, 1982; Biely, 1985; Grant and Rhodes, 1992; Confer and Logan, 1998; Lenartovicz et al., 2003; Collins et al., 2005). As the hyphal growth has been highlighted as an important characteristic to colonize substrates (Zalar et al., 2005), *B. parvus* might be using cell-attached enzymes to hydrolytic cleave them.

In marine environments, as bacteria and fungi might have similar functions like decomposers and their close spatial proximity, might lead to antagonistic or synergistic interactions (Velicer, 2003; Romaní et al., 2006). In marine bacteria, the direct release of cell-free enzymes has been reported as a response to the presence of substrate (Alderkamp et al., 2007), starvation (Albertson et al., 1990; Alderkamp et al., 2007; Bong et al., 2013), changes in the cell permeability (Chróst, 1990), or due to cell decay (Baltar et al., 2019). However, when the substrate becomes limited, but the secretion of cell-free enzymes by marine fungi remains unknown. In freshwater studies, Millar et al. (2015) reported contributions of microbial cell-free enzymatic activities of 15.5, 32.6, 32.9, 82.5, and 24.4% for BGL, NAG, APA, SUL, and LAP, respectively, of the total EEA. In contrast, Kamer and Rassoulzadegan (1995) and Baltar (2018) suggested that the marine microbial cell-free fraction can represent up to 100% of the total EEA. These authors reported microbial cell-free enzymatic activities ranging from 0, 37, and 34% for BGL, APA, and LAP, respectively, and up to 100% of the total EEA (Baltar et al., 2010; Baltar et al., 2013; Baltar et al., 2016). In the present study, we report that depending on the species and also on the salinity, the fungal cell-free fraction can vary from 0.1 to 85.1% of the total EEA with some enzymes more likely to produce cell-free enzymes like OLE and SUL.

### 4.3 Potential environmental implications

Climate change is influencing the freshwater input from ice, riverine, and precipitation into oceans, hence, also its salinity (Myers et al., 1990; Munk, 2003; Reid et al., 2009; Hutchins and Fu, 2017; Kohler et al., 2020). The desalination of the oceans, also known as ocean freshening, can intensify the stratification which decreases the vertical mixing of the water column and affects the transport of nutrients (Reid et al., 2009; Balaguru et al., 2016; Hutchins and Fu, 2017). As suggested by Arnosti et al. (2014), nutrient availability can influence the magnitude and distribution of extracellular enzymatic activities. Accordingly, a different nutrient availability might lead to expression of other enzymes, which might also influence the potential role of marine fungi in the oceanic biogeochemical cycles. Moreover, most marine fungi are adapted to tolerate high salinity (Jennings, 1983), so salinity can influence the fungal community composition (Rojas-Jimenez et al., 2019). Our results indicate that the studied marine fungal species might be adapted to different salinities, but their extracellular enzymatic activities, both, cell-attached and cell-free, might be affected by salinity.

## 5 Conclusions

Based on our results, the marine fungi species studied are capable to secrete an array of cell-free enzymes, and this can represent up to 85.1% of the respective total EEA. Though, it is important to consider that some of these high values were found in fungal strains and enzymes with low total EEA, so their contribution to the cell-free enzymatic pool might be minimal compared to others. Additionally, the release of these extracellular enzymes can be influenced by environmental parameters such as salinity, despite being species-specific. As fungi are undeniably widespread in marine environments, their cell-free enzymes might also be an important part of the oceanic enzymatic pool. Nonetheless, as oceans are complex and diverse ecosystems, other abiotic and biotic variables should also be included in future studies.

## Data availability statement

The raw data supporting the conclusions of this article will be made available by the authors, without undue reservation.

## Author contributions

Conceptualization, KS-A, and FB; data curation, KS-A; formal analysis, KS-A; funding acquisition, FB; investigation, KS-A; methodology, KS-A, and FB; project administration, FB; resources, GH, and FB; software, KS-A; supervision, FB; validation, KS-A; visualization, KS-A; writing original draft, KS-A; writing, review, and editing, KS-A, GH, and FB. All authors have read and agreed to the published version of the manuscript.

## References

[B1] Albertson N. H. Nyström T. Kjelleberg S. (1990). Exoprotease activity of two marine bacteria during starvation. Appl. Environ. Microbiol. 56, 218–223. doi: 10.1128/aem.56.1.218-223.1990 16348094 PMC183279

[B2] Alderkamp A.-C. Van Rijssel M. Bolhuis H. (2007). Characterization of marine bacteria and the activity of their enzyme systems involved in degradation of the algal storage glucan laminarin. FEMS Microbiol. Ecol. 59, 108–117. doi: 10.1111/j.1574-6941.2006.00219.x 17233748

[B3] Allen T. W. Quayyum H. A. Burpee L. L. Buck J. W. (2004). Effect of foliar disease on the epiphytic yeast communities of creeping bentgrass and tall fescue. Can. J. Microbiol. 50, 853–860. doi: 10.1139/w04-073 15644900

[B4] Allison S. D. (2005). Cheaters, diffusion and nutrients constrain decomposition by microbial enzymes in spatially structured environments. Ecol. Lett. 8, 626–635. doi: 10.1111/j.1461-0248.2005.00756.x

[B5] Allison S. D. Chao Y. Farrara J. D. Hatosy S. Martiny A. C. (2012). Fine-scale temporal variation in marine extracellular enzymes of coastal southern California. Front. Microbiol. 3, 1–10. doi: 10.3389/fmicb.2012.00301 22912628 PMC3421452

[B6] Amend A. Burgaud G. Cunliffe M. Edgcomb V. P. Ettinger C. L. Gutiérrez M. H.. (2019). Fungi in the marine environment: open questions and unsolved problems. mBio 10, 1–18. doi: 10.1128/mBio.01189-18 PMC6401481 30837337

[B7] Amon R. M. Benner R. (1994). Rapid cycling of high-molecular-weight dissolved organic matter in the ocean. Nature 369, 549–552. doi: 10.1038/369549a0

[B8] Amon R. M. Benner R. (1996). Bacterial utilization of different size classes of dissolved organic matter. Limnol. Oceanogr. 41, 41–51. doi: 10.4319/lo.1996.41.1.0041

[B9] Amon R. M. Benner R. (2003). Combined neutral sugars as indicators of the diagenetic state of dissolved organic matter in the Arctic Ocean. Deep Sea Res. Part I: Oceanograph. Res. Papers 50, 151–169. doi: 10.1016/S0967-0637(02)00130-9

[B10] Aquino R. S. Grativol C. Mourão P. A. (2011). Rising from the sea: correlations between sulfated polysaccharides and salinity in plants. PloS One 6, 1–11. doi: 10.1371/journal.pone.0018862 PMC3084243 21552557

[B11] Aquino R. S. Landeira-Fernandez A. M. Valente A. P. Andrade L. R. Mourao P. A. (2005). Occurrence of sulfated galactans in marine angiosperms: evolutionary implications. Glycobiology 15, 11–20. doi: 10.1093/glycob/cwh138 15317737

[B12] Arfi Y. Chevret D. Henrissat B. Berrin J.-G. Levasseur A. Record E. (2013). Characterization of salt-adapted secreted lignocellulolytic enzymes from the mangrove fungus Pestalotiopsis sp. Nat. Commun. 4, 1–9. doi: 10.1038/ncomms2850 23651998

[B13] Arnosti C. (2011). Microbial extracellular enzymes and the marine carbon cycle. Annu. Rev. Mar. Sci. 3, 401–425. doi: 10.1146/annurev-marine-120709-142731 21329211

[B14] Arnosti C. Bell C. Moorhead D. L. Sinsabaugh R. L. Steen A. D. Stromberger M.. (2014). Extracellular enzymes in terrestrial, freshwater, and marine environments: perspectives on system variability and common research needs. Biogeochemistry 117, 5–21. doi: 10.1007/s10533-013-9906-5

[B15] Balaguru K. Foltz G. R. Leung L. R. Emanuel K. A. (2016). Global warming-induced upper-ocean freshening and the intensification of super typhoons. Nat. Commun. 7, 1–8. doi: 10.1038/ncomms13670 PMC5133693 27886199

[B16] Baltar F. (2018). Watch out for the “living dead”: cell-free enzymes and their fate. Front. Microbiol. 8, 1–6. doi: 10.3389/fmicb.2017.02438 PMC5758490 29354095

[B17] Baltar F. Arístegui J. Gasol J. M. Sintes E. Van Aken H. M. Herndl G. J. (2010). High dissolved extracellular enzymatic activity in the deep central Atlantic Ocean. Aquat. Microbial Ecol. 58, 287–302. doi: 10.3354/ame01377

[B18] Baltar F. Arístegui J. Gasol J. M. Yokokawa T. Herndl G. J. (2013). Bacterial versus archaeal origin of extracellular enzymatic activity in the Northeast Atlantic deep waters. Microbial Ecol. 65, 277–288. doi: 10.1007/s00248-012-0126-7 23015014

[B19] Baltar F. De Corte D. Yokokawa T. (2019). Bacterial stress and mortality may be a source of cell-free enzymatic activity in the marine environment. Microbes Environ. 34, 83–88. doi: 10.1264/jsme2.ME18123 30799317 PMC6440733

[B20] Baltar F. Legrand C. Pinhassi J. (2016). Cell-free extracellular enzymatic activity is linked to seasonal temperature changes: a case study in the Baltic Sea. Biogeosciences 13, 2815–2821. doi: 10.5194/bg-13-2815-2016

[B21] Baltar F. Zhao Z. Herndl G. J. (2021). Potential and expression of carbohydrate utilization by marine fungi in the global ocean. Microbiome 9, 1–10. doi: 10.1186/s40168-021-01063-4 33975640 PMC8114511

[B22] Baxter R. (1959). An interpretation of the effects of salts on the lactic dehydrogenase of Halobacterium salinarium. Can. J. Microbiol. 5, 47–57. doi: 10.1139/m59-006 13629384

[B23] Benner R. (2002). Chemical composition and reactivity. Biogeochemistry Mar. Dissolved Organic Matter 1, 59–90. doi: 10.1016/B978-012323841-2/50005-1

[B24] Benner R. Pakulski J. D. McCarthy M. Hedges J. I. Hatcher P. G. (1992). Bulk chemical characteristics of dissolved organic matter in the ocean. Science 255, 1561–1564. doi: 10.1126/science.255.5051.1561 17820170

[B25] Bergé J.-P. Barnathan G. (2005). Fatty acids from lipids of marine organisms: molecular biodiversity, roles as biomarkers, biologically active compounds, and economical aspects. Mar. Biotechnol. 1, 49–125. doi: 10.1007/b135782 16566089

[B26] Bhat K. M. Gaikwad J. S. Maheshwari R. (1993). Purification and characterization of an extracellular β-glucosidase from the thermophilic fungus Sporotrichum thermophile and its influence on cellulase activity. Microbiology 139, 2825–2832. doi: 10.1099/00221287-139-11-2825

[B27] Biely P. (1985). Microbial xylanolytic systems. Trends Biotechnol. 3, 286–290. doi: 10.1016/0167-7799(85)90004-6

[B28] Bong C. W. Obayashi Y. Suzuki S. (2013). Succession of protease activity in seawater and bacterial isolates during starvation in a mesocosm experiment. Aquat. Microbial Ecol. 69, 33–46. doi: 10.3354/ame01618

[B29] Brembu T. Mühlroth A. Alipanah L. Bones A. M. (2017). The effects of phosphorus limitation on carbon metabolism in diatoms. Philos. Trans. R. Soc. London. Ser. B: Biol. Sci. 372, 1–10. doi: 10.1098/rstb.2016.0406 PMC5516115 28717016

[B30] Caruso G. Zaccone R. (2000). Estimates of leucine aminopeptidase activity in different marine and brackish environments. J. Appl. Microbiol. 89, 951–959. doi: 10.1046/j.1365-2672.2000.01198.x 11123468

[B31] Celussi M. Del Negro P. (2012). Microbial degradation at a shallow coastal site: long-term spectra and rates of exoenzymatic activities in the NE Adriatic Sea. Estuarine Coast. Shelf Sci. 115, 75–86. doi: 10.1016/j.ecss.2012.02.002

[B32] Chandrasekaran M. Kumar S. R. (1997). Marine microbial enzymes. Biotechnology 9, 47–79.

[B33] Chróst R. J. (1986). “Algal-bacterial metabolic coupling in the carbon and phosphorus cycle in lakes,” in Proc. IV ISME, Vol. 271. 360–366.

[B34] Chróst R. J. (1990). “Microbial ectoenzymes in aquatic environments,” in Aquatic microbial ecology. Eds. Overbeck J. Chróst R. J. (New York: Springer), 47–78.

[B35] Chróst R. J. (1992). Significance of bacterial ectoenzymes in aquatic environments. Hydrobiologia 243, 61–70. doi: 10.1007/BF00007020

[B36] Chróst R. J. Overbeck J. (1987). Kinetics of alkaline phosphatase activity and phosphorus availability for phytoplankton and bacterioplankton in lake plu\see (North German Eutrophic Lake). Microbial Ecol. 13, 229–248. doi: 10.1007/BF02025000 24213298

[B37] Chróst R. J. Rai H. (1993). Ectoenzyme activity and bacterial secondary production in nutrient-impoverished and nutrient-enriched freshwater mesocosms. Microbial Ecol. 25, 131–150. doi: 10.1007/BF00177191 24189811

[B38] Chrost R. J. Siuda W. HALEMEjko G. Z. (1984). Longterm studies on alkaline phosphatase activity (APA) in a lake with fish-aquaculture in relation to lake eutrophication and phosphorus cycle. Archiv für Hydrobiologie 70, 1–32.

[B39] Ciancia M. Fernández P. V. Leliaert F. (2020). Diversity of sulfated polysaccharides from cell walls of coenocytic green algae and their structural relationships in view of green algal evolution. Front. Plant Sci. 11, 1–15. doi: 10.3389/fpls.2020.554585 33133113 PMC7550628

[B40] Collins T. Gerday C. Feller G. (2005). Xylanases, xylanase families and extremophilic xylanases. FEMS Microbiol. Rev. 29, 3–23. doi: 10.1016/j.femsre.2004.06.005 15652973

[B41] Colman A. S. Blake R. E. Karl D. M. Fogel M. L. Turekian K. K. (2005). Marine phosphate oxygen isotopes and organic matter remineralization in the oceans. Proc. Natl. Acad. Sci. 102, 13023–13028. doi: 10.1073/pnas.0506455102 16141319 PMC1201620

[B42] Confer D. R. Logan B. E. (1998). Location of protein and polysaccharide hydrolytic activity in suspended and biofilm wastewater cultures. Water Res. 32, 31–38. doi: 10.1016/S0043-1354(97)00194-2

[B43] D’ambrosio L. Ziervogel K. MacGregor B. Teske A. Arnosti C. (2014). Composition and enzymatic function of particle-associated and free-living bacteria: a coastal/offshore comparison. ISME J. 8, 2167–2179. doi: 10.1038/ismej.2014.67 24763371 PMC4992077

[B44] Defaye J. Guillot J.-M. Biely P. Vršanská M. (1992). Positional isomers of thioxylobiose, their synthesis and inducing ability for D-xylan-degrading enzymes in the yeast Cryptococcus albidus. Carbohydr. Res. 228, 47–64. doi: 10.1016/S0008-6215(00)90548-2 1516094

[B45] Dix N. J. Webster J. (1995). “Fungi of extreme environments,” in Fungal ecology. Eds. Dix N. J. Webster J. (Dordrecht: Springer), 322–340.

[B46] Duarte A. W. F. Bonugli-Santos R. C. Duarte A. L. F. Gomes E. Sette L. D. (2021). Statistical experimental design applied to extracellular lipase production by the marine Antarctic yeast Leucosporidium scottii CRM 728. Biocatalysis Agric. Biotechnol. 32, 1–8. doi: 10.1016/j.bcab.2021.101954

[B47] Dubovenko A. G. Dunaevsky Y. E. Belozersky M. A. Oppert B. Lord J. C. Elpidina E. N. (2010). Trypsin-like proteins of the fungi as possible markers of pathogenicity. Fungal Biol. 114, 151–159. doi: 10.1016/j.funbio.2009.11.004 20960971

[B48] Duhamel S. Dyhrman S. T. Karl D. M. (2010). Alkaline phosphatase activity and regulation in the North Pacific Subtropical Gyre. Limnology Oceanography 55, 1414–1425. doi: 10.4319/lo.2010.55.3.1414

[B49] Edson C. Brody S. (1976). Biochemical and genetic studies on galactosamine metabolism in Neurospora crassa. J. Bacteriology 126, 799–805. doi: 10.1128/jb.126.2.799-805.1976 PMC233216 131123

[B50] Elyas K. Mathew A. Sukumaran R. K. Ali P. M. Sapna K. Kumar S. R.. (2010). Production optimization and properties of beta glucosidases from a marine fungus Aspergillus-SA 58. New Biotechnol. 27, 347–351. doi: 10.1016/j.nbt.2010.02.007 20219710

[B51] Fell J. W. Hunter I. L. (1968). Isolation of heterothallic yeast strains of Metschnikowia Kamienski and their mating reactions with Chlamydozyma Wickerham spp. Antonie van Leeuwenhoek 34, 365–376. doi: 10.1007/BF02046459 5305789

[B52] Fell J. W. Hunter I. L. Tallman A. S. (1973). Marine basidiomycetous yeasts (Rhodosporidium spp. n.) with tetrapolar and multiple allelic bipolar mating systems. Can. J. Microbiol. 19, 643–657. doi: 10.1139/m73-106 4575449

[B53] Fell J. W. Statzell A. C. (1971). Sympodiomyces gen. n., a yeast-like organism from southern marine waters. Antonie van Leeuwenhoek 37, 359–367. doi: 10.1007/BF02218506 5315727

[B54] Fickers P. Fudalej F. Le Dall M.-T. Casaregola S. Gaillardin C. Thonart P.. (2005). Identification and characterisation of LIP7 and LIP8 genes encoding two extracellular triacylglycerol lipases in the yeast Yarrowia lipolytica. Fungal Genet. Biol. 42, 264–274. doi: 10.1016/j.fgb.2004.12.003 15707847

[B55] Francis M. Webb V. Zuccarello G. (2016). Marine yeast biodiversity on seaweeds in New Zealand waters. New Z. J. Bot. 54, 30–47. doi: 10.1080/0028825X.2015.1103274

[B56] Gadanho M. Almeida J. M. Sampaio J. P. (2003). Assessment of yeast diversity in a marine environment in the south of Portugal by microsatellite-primed PCR. Antonie van Leeuwenhoek 84, 217–227. doi: 10.1023/A:1026038213195 14574117

[B57] Gaderer R. Seidl-Seiboth V. de Vries R. P. Seiboth B. Kappel L. (2017). N-acetylglucosamine, the building block of chitin, inhibits growth of Neurospora crassa. Fungal Genet. Biol. 107, 1–11. doi: 10.1016/j.fgb.2017.07.005 28736299

[B58] Gaikwad J. S. Maheshwari R. (1994). Localization and release of β-glucosidase in the thermophilic and cellulolytic fungus, Sporotrichum thermophile. Exp. Mycology 18, 300–310. doi: 10.1016/S0147-5975(06)80003-4

[B59] Garcia-Rubio R. de Oliveira H. C. Rivera J. Trevijano-Contador N. (2020). The fungal cell wall: Candida, Cryptococcus, and Aspergillus species. Front. Microbiol. 10, 1–13. doi: 10.3389/fmicb.2019.02993 PMC6962315 31993032

[B60] Gladfelter A. S. James T. Y. Amend A. S. (2019). Marine fungi. Curr. Biol. 29, 191–195. doi: 10.1016/j.cub.2019.02.009 30889385

[B61] Gonçalves M. F. Hilário S. Van de Peer Y. Esteves A. C. Alves A. (2021). Genomic and metabolomic analyses of the marine fungus Emericellopsis cladophorae: insights into saltwater adaptability mechanisms and its biosynthetic potential. J. Fungi 8, 1–20. doi: 10.3390/jof8010031 PMC8780691 35049971

[B62] Grant W. Rhodes L. (1992). Cell-bound and extracellular laminarinase activity in Dendryphiella salina and five other marine fungi. Botanica Marina 1, 503–511. doi: 10.1515/botm.1992.35.6.503

[B63] Gupta R. Beg Q. Lorenz P. (2002). Bacterial alkaline proteases: molecular approaches and industrial applications. Appl. Microbiol. Biotechnol. 59, 15–32. doi: 10.1007/s00253-002-0975-y 12073127

[B64] Gutiérrez M. H. Pantoja S. Tejos E. Quiq ones R. A. (2011). The role of fungi in processing marine organic matter in the upwelling ecosystem off Chile. Mar. Biol. 158, 205–219. doi: 10.1007/s00227-010-1552-z

[B65] Hartl L. Zach S. Seidl-Seiboth V. (2012). Fungal chitinases: diversity, mechanistic properties and biotechnological potential. Appl. Microbiol. Biotechnol. 93, 533–543. doi: 10.1007/s00253-011-3723-3 22134638 PMC3257436

[B66] Hassan H. Pratt D. (1977). Biochemical and physiological properties of alkaline phosphatases in five isolates of marine bacteria. J. Bacteriology 129, 1607–1612. doi: 10.1128/jb.129.3.1607-1612.1977 PMC235141 845125

[B67] Helbert W. (2017). Marine polysaccharide sulfatases. Front. Mar. Sci. 4, 1–10. doi: 10.3389/fmars.2017.00006

[B68] Hettle A. G. Vickers C. J. Boraston A. B. (2022). Sulfatases: critical enzymes for algal polysaccharide processing. Front. Plant Sci. 13, 1–12. doi: 10.3389/fpls.2022.837636 PMC9096561 35574087

[B69] Hollibaugh J. Azam F. (1983). Microbial degradation of dissolved proteins in seawater. Limnology Oceanography 28, 1104–1116. doi: 10.4319/lo.1983.28.6.1104

[B70] Hoppe H.-G. (1983). Significance of exoenzymatic activities in the ecology of brackish water: measurements by means of methylumbelliferyl-substrates. Mar. Ecol. Prog. Ser. 11, 299–308. doi: 10.3354/meps011299

[B71] Hoppe H.-G. Arnosti C. Herndl G. J. (2002). Ecological significance of bacterial enzymes in the marine environment. Enzymes Environ.: Activity Eco. Appl. 1, 73–107. doi: 10.1201/9780203904039.ch3

[B72] Hoppe H.-G. Ullrich S. (1999). Profiles of ectoenzymes in the Indian Ocean: phenomena of phosphatase activity in the mesopelagic zone. Aquat. Microbial Ecol. 19, 139–148. doi: 10.3354/ame019139

[B73] Huitema C. Horsman G. (2018). Analyzing enzyme kinetic data using the powerful statistical capabilities of R. bioRxiv 1, 1–12. doi: 10.1101/316588

[B74] Hutcheon G. W. Vasisht N. Bolhuis A. (2005). Characterisation of a highly stable α-amylase from the halophilic archaeon Haloarcula hispanica. Extremophiles 9, 487–495. doi: 10.1007/s00792-005-0471-2 16075161

[B75] Hutchins D. A. Fu F. (2017). Microorganisms and ocean global change. Nat. Microbiol. 2, 1–11. doi: 10.1038/nmicrobiol.2017.58 28540925

[B76] Jennings D. (1983). Some aspects of the physiology and biochemistry of marine fungi. Biol. Rev. 58, 423–459. doi: 10.1111/j.1469-185X.1983.tb00395.x

[B77] Jin M. Gai Y. Guo X. Hou Y. Zeng R. (2019). Properties and applications of extremozymes from deep-sea extremophilic microorganisms: a mini review. Mar. Drugs 17, 1–16. doi: 10.3390/md17120656 PMC6950199 31766541

[B78] Kamer M. Rassoulzadegan F. (1995). Extracellular enzyme activity: indications for high short-term variability in a coastal marine ecosystem. Microbial Ecol. 30, 143–156. doi: 10.1007/BF00172570 24185481

[B79] Karan R. Capes M. D. DasSarma S. (2012). Function and biotechnology of extremophilic enzymes in low water activity. Aquat. Biosyst. 8, 1–15. doi: 10.1186/2046-9063-8-4 22480329 PMC3310334

[B80] Karan R. Mathew S. Muhammad R. Bautista D. B. Vogler M. Eppinger J.. (2020). Understanding high-salt and cold adaptation of a polyextremophilic enzyme. Microorganisms 8, 1–19. doi: 10.3390/microorganisms8101594 PMC7602713 33081237

[B81] Karner M. Herndl G. J. (1992). Extracellular enzymatic activity and secondary production in free-living and marine-snow-associated bacteria. Mar. Biol. 113, 341–347. doi: 10.1007/BF00347289

[B82] Keith S. Arnosti C. (2001). Extracellular enzyme activity in a river-bay-shelf transect: variations in polysaccharide hydrolysis rates with substrate and size class. Aquat. Microbial Ecol. 24, 243–253. doi: 10.3354/ame024243

[B83] Kim C. Nishimura Y. Nagata T. (2007). High potential activity of alkaline phosphatase in the benthic nepheloid layer of a large mesotrophic lake: implications for phosphorus regeneration in oxygenated hypolimnion. Aquat. Microbial Ecol. 49, 303–311. doi: 10.3354/ame01137

[B84] King G. M. (1986). Characterization of β-glucosidase activity in intertidal marine sediments. Appl. Environ. Microbiol. 51, 373–380. doi: 10.1128/aem.51.2.373-380.1986 16346994 PMC238876

[B85] Kiran G. S. Hema T. Gandhimathi R. Selvin J. Thomas T. A. Ravji T. R.. (2009). Optimization and production of a biosurfactant from the sponge-associated marine fungus Aspergillus ustus MSF3. Colloids Surfaces B: Biointerf. 73, 250–256. doi: 10.1016/j.colsurfb.2009.05.025 19570659

[B86] Kloareg B. Quatrano R. (1988). Structure of the cell walls of marine algae and ecophysiological functions of the matrix polysaccharides. Oceanogr Mar. Biol. Annu. Rev. 26, 259–315.

[B87] Klotz M. G. Bryant D. A. Hanson T. E. (2011). The microbial sulfur cycle. Front. Microbiol. 2, 1–2. doi: 10.3389/fmicb.2011.00241 22144979 PMC3228992

[B88] Kohler T. J. Peter H. Fodelianakis S. Pramateftaki P. Styllas M. Tolosano M.. (2020). Patterns and drivers of extracellular enzyme activity in New Zealand glacier-fed streams. Front. Microbiol. 11, 1–14. doi: 10.3389/fmicb.2020.591465 33329472 PMC7711088

[B89] Kornblatt J. Kornblatt M. (2002). Water as it applies to the function of enzymes. Int. Rev. Cytology 215, 49–73. doi: 10.1016/S0074-7696(02)15005-4 11952237

[B90] Kubicek C. P. Herrera-Estrella A. Seidl-Seiboth V. Martinez D. A. Druzhinina I. S. Thon M.. (2011). Comparative genome sequence analysis underscores mycoparasitism as the ancestral life style of Trichoderma. Genome Biol. 12, 1–15. doi: 10.1186/gb-2011-12-4-r40 PMC3218866 21501500

[B91] Kuntz I. D. Jr (1971). Hydration of macromolecules. IV. Polypeptide conformation in frozen solutions. J. Am. Chem. Soc. 93, 516–518. doi: 10.1016/0003-9861(71)90532-7 5541519

[B92] Lanyi J. K. (1974). Salt-dependent properties of proteins from extremely halophilic bacteria. Bacteriolog. Rev. 38, 272–290. doi: 10.1128/br.38.3.272-290.1974 PMC413857 4607500

[B93] Larsen H. (1967). “Biochemical aspects of extreme halophilism,” in Advances in microbial physiology. Eds. Rose A. H. Wilkinson J. F. (Cambridge: Academic Press), 97–132.

[B94] Lee S. Park M. S. Lee H. Kim J.-J. Eimes J. A. Lim Y. W. (2019). Fungal diversity and enzyme activity associated with the macroalgae, Agarum clathratum. Mycobiology 47, 50–58. doi: 10.1080/12298093.2019.1580464 31001450 PMC6452909

[B95] Lenartovicz V. de Souza C. G. M. Moreira F. G. Peralta R. M. (2003). Temperature and carbon source affect the production and secretion of a thermostable β-xylosidase by Aspergillus fumigatus. Process Biochem. 38, 1775–1780. doi: 10.1016/S0032-9592(02)00261-3

[B96] Li X. Kondo R. Sakai K. (2002). Studies on hypersaline-tolerant white-rot fungi L: screening of lignin-degrading fungi in hypersaline conditions. J. Wood Sci. 48, 147–152. doi: 10.1007/BF00767292

[B97] Li Y. Sun L.-L. Sun Y.-Y. Cha Q.-Q. Li C.-Y. Zhao D.-L.. (2019). Extracellular enzyme activity and its implications for organic matter cycling in northern Chinese marginal seas. Front. Microbiol. 10, 1–13. doi: 10.3389/fmicb.2019.02137 31608022 PMC6755343

[B98] Lockwood S. Greening C. Baltar F. Morales S. E. (2022). Global and seasonal variation of marine phosphonate metabolism. ISME J. 16, 1–15. doi: 10.1038/s41396-022-01266-z 35739297 PMC9381506

[B99] Louhasakul Y. Cheirsilp B. Prasertsan P. (2016). Valorization of palm oil mill effluent into lipid and cell-bound lipase by marine yeast Yarrowia lipolytica and their application in biodiesel production. Waste Biomass Valorization 7, 417–426. doi: 10.1007/s12649-015-9451-7

[B100] Luo H. Benner R. Long R. A. Hu J. (2009). Subcellular localization of marine bacterial alkaline phosphatases. Proc. Natl. Acad. Sci. 106, 21219–21223. doi: 10.1073/pnas.0907586106 19926862 PMC2795515

[B101] MacDonald M. J. Hartley D. L. Speedie M. K. (1985). Location of cellulolytic enzyme activity in the marine fungus Trichocladium achrasporum. Can. J. Microbiol. 31, 145–148. doi: 10.1139/m85-028

[B102] MacDonald M. J. Speedie M. K. (1982). Cell-associated and extracellular cellulolytic enzyme activity in the marine fungus Dendryphiella arenaria. Can. J. Bot. 60, 838–844. doi: 10.1139/b82-107

[B103] Mancheño J. M. Pernas M. A. A. Martıñez M. A. J. Ochoa B. Rúa M. L. Hermoso J. A. (2003). Structural insights into the lipase/esterase behavior in the Candida rugosa lipases family: crystal structure of the lipase 2 isoenzyme at 1.97 Å resolution. J. Mol. Biol. 332, 1059–1069. doi: 10.1016/j.jmb.2003.08.005 14499609

[B104] Matsumoto Y. Saucedo-Castañeda G. Revah S. Shirai K. (2004). Production of β-N-acetylhexosaminidase of Verticillium lecanii by solid state and submerged fermentations utilizing shrimp waste silage as substrate and inducer. Process Biochem. 39, 665–671. doi: 10.1016/S0032-9592(03)00140-7

[B105] McCarthy M. Hedges J. Benner R. (1996). Major biochemical composition of dissolved high molecular weight organic matter in seawater. Mar. Chem. 55, 281–297. doi: 10.1016/S0304-4203(96)00041-2

[B106] Millar J. J. Payne J. T. Ochs C. A. Jackson C. R. (2015). Particle-associated and cell-free extracellular enzyme activity in relation to nutrient status of large tributaries of the Lower Mississippi River. Biogeochemistry 124, 255–271. doi: 10.1007/s10533-015-0096-1

[B107] Morales S. E. Biswas A. Herndl G. J. Baltar F. (2019). Global structuring of phylogenetic and functional diversity of pelagic fungi by depth and temperature. Front. Mar. Sci. 6, 1–12. doi: 10.3389/fmars.2019.00131 36817748

[B108] Munk W. (2003). Ocean freshening, sea level rising. Science 300, 2041–2043. doi: 10.1126/science.1085534 12829770

[B109] Muszewska A. Stepniewska-Dziubinska M. M. Steczkiewicz K. Pawlowska J. Dziedzic A. Ginalski K. (2017). Fungal lifestyle reflected in serine protease repertoire. Sci. Rep. 7, 1–12. doi: 10.1038/s41598-017-09644-w 28831173 PMC5567314

[B110] Myers R. A. Akenhead S. A. Drinkwater K. (1990). The influence of Hudson Bay runoff and ice-melt on the salinity of the inner Newfoundland Shelf. Atmosphere-Ocean 28, 241–256. doi: 10.1080/07055900.1990.9649377

[B111] Newell S. Y. Fell J. W. (1970). The perfect form of a marine-occurring yeast of the genus Rhodotorula. Mycologia 62, 272–281. doi: 10.1080/00275514.1970.12018965

[B112] Obayashi Y. Suzuki S. (2008a). Adsorption of extracellular proteases in seawater onto filters during size fractionation. J. Oceanography 64, 367–372. doi: 10.1007/s10872-008-0029-x

[B113] Obayashi Y. Suzuki S. (2008b). Occurrence of exo-and endopeptidases in dissolved and particulate fractions of coastal seawater. Aquat. Microbial Ecol. 50, 231–237. doi: 10.3354/ame01169

[B114] Ohta K. Fujimoto H. Fujii S. Wakiyama M. (2010). Cell-associated β-xylosidase from Aureobasidium pullulans ATCC 20524: purification, properties, and characterization of the encoding gene. J. Bioscience Bioengineering 110, 152–157. doi: 10.1016/j.jbiosc.2010.02.008 20547381

[B115] Pantoja S. Lee C. (1999). Peptide decomposition by extracellular hydrolysis in coastal seawater and salt marsh sediment. Mar. Chem. 63, 273–291. doi: 10.1016/S0304-4203(98)00067-X

[B116] Pantoja S. Lee C. Marecek J. F. (1997). Hydrolysis of peptides in seawater and sediment. Mar. Chem. 57, 25–40. doi: 10.1016/S0304-4203(97)00003-0

[B117] Papanikolaou S. Chevalot I. Galiotou-Panayotou M. Komaitis M. Marc I. Aggelis G. (2007). Industrial derivative of tallow: a promising renewable substrate for microbial lipid, single-cell protein and lipase production by Yarrowia lipolytica. Electronic J. Biotechnol. 10, 425–435. doi: 10.2225/vol10-issue3-fulltext-8

[B118] Parawira W. Murto M. Read J. Mattiasson B. (2005). Profile of hydrolases and biogas production during two-stage mesophilic anaerobic digestion of solid potato waste. Process Biochem. 40, 2945–2952. doi: 10.1016/j.procbio.2005.01.010

[B119] Parrish C. C. (2013). Lipids in marine ecosystems. Int. Scholarly Res. Notices 1, 1–16. doi: 10.5402/2013/604045

[B120] Paytan A. McLaughlin K. (2007). The oceanic phosphorus cycle. Chem. Rev. 107, 563–576. doi: 10.1021/cr0503613 17256993

[B121] Pointing S. Buswell J. Jones E. G. Vrijmoed L. (1999). Extracellular cellulolytic enzyme profiles of five lignicolous mangrove fungi. Mycological Res. 103, 696–700. doi: 10.1017/S0953756298007655

[B122] Priest F. G. (1977). Extracellular enzyme synthesis in the genus Bacillus. Bacteriological Rev. 41, 711–753. doi: 10.1128/br.41.3.711-753.1977 PMC414021 334155

[B123] Raghukumar S. (2017). Fungi in coastal and oceanic marine ecosystems (Cham: Springer).

[B124] Reese E. Maguire A. Parrish F. (1973). Production of β-D-xylopyranosidases by fungi. Can. J. Microbiol. 19, 1065–1074. doi: 10.1139/m73-170 4754745

[B125] Rego J. V. Billen G. Fontigny A. Somville M. (1985). Free and attached proteolytic activity in water environments. Mar. Ecol. Prog. Ser. 21, 245–249. doi: 10.3354/meps021245

[B126] Reid P. C. Fischer A. C. Lewis-Brown E. Meredith M. P. Sparrow M. Andersson A. J.. (2009). Impacts of the oceans on climate change. Adv. Mar. Biol. 56, 1–150. doi: 10.1016/S0065-2881(09)56001-4 19895974

[B127] Repeta D. J. (2015). “Chemical characterization and cycling of dissolved organic matter,” in Biogeochemistry of marine dissolved organic matter. Eds. Hansell D. A. Carlson C. A. (Cambridge: Academic Press), 21–63.

[B128] Rezaei K. Jenab E. Temelli F. (2007). Effects of water on enzyme performance with an emphasis on the reactions in supercritical fluids. Crit. Rev. Biotechnol. 27, 183–195. doi: 10.1080/07388550701775901 18085461

[B129] Richards T. A. Jones M. D. Leonard G. Bass D. (2012). Marine fungi: their ecology and molecular diversity. Annu. Rev. Mar. Sci. 4, 495–522. doi: 10.1146/annurev-marine-120710-100802 22457985

[B130] Richards T. A. Talbot N. J. (2013). Horizontal gene transfer in osmotrophs: playing with public goods. Nat. Rev. Microbiol. 11, 720–727. doi: 10.1038/nrmicro3108 24018383

[B131] Rojas-Jimenez K. Rieck A. Wurzbacher C. Jürgens K. Labrenz M. Grossart H.-P. (2019). A salinity threshold separating fungal communities in the Baltic Sea. Front. Microbiol. 10, 1–9. doi: 10.3389/fmicb.2019.00680 30984159 PMC6449873

[B132] Romaní A. M. Fischer H. Mille-Lindblom C. Tranvik L. J. (2006). Interactions of bacteria and fungi on decomposing litter: differential extracellular enzyme activities. Ecology 87, 2559–2569. doi: 10.1890/0012-9658(2006)87[2559:IOBAFO]2.0.CO;2 17089664

[B133] Ruiz-Herrera J. (1991). Fungal cell wall: structure, synthesis, and assembly (Boca Ratón: CRC Press).

[B134] Saenger W. (1987). Structure and dynamics of water surrounding biomolecules. Annu. Rev. Biophysics Biophys. Chem. 16, 93–114. doi: 10.1146/annurev.bb.16.060187.000521 3297093

[B135] Salazar-Alekseyeva K. Herndl G. J. Baltar F. (2023). Influence of salinity on the extracellular enzymatic activity of marine pelagic fungi. 1–22. 10.3390/jof10020152 PMC10890631 38392824

[B136] Salazar Alekseyeva K. Herndl G. J. Baltar F. (2022). Extracellular enzymatic activities of oceanic pelagic fungal strains and the influence of temperature. J. Fungi 8, 1–17. doi: 10.3390/jof8060571 PMC9225461 35736054

[B137] Salazar Alekseyeva K. Mähnert B. Berthiller F. Breyer E. Herndl G. J. Baltar F. (2021). Adapting an ergosterol extraction method with marine yeasts for the quantification of oceanic fungal biomass. J. Fungi 7, 1–12. doi: 10.3390/jof7090690 PMC8468844 34575728

[B138] Schultz-Johansen M. Bech P. K. Hennessy R. C. Glaring M. A. Barbeyron T. Czjzek M.. (2018). A novel enzyme portfolio for red algal polysaccharide degradation in the marine bacterium Paraglaciecola hydrolytica S66T encoded in a sizeable polysaccharide utilization locus. Front. Microbiol. 9, 1–15. doi: 10.3389/fmicb.2018.00839 29774012 PMC5943477

[B139] Scioli C. Vollaro L. (1997). The use of Yarrowia lipolytica to reduce pollution in olive mill wastewaters. Water Res. 31, 2520–2524. doi: 10.1016/S0043-1354(97)00083-3

[B140] Scott W. A. Metzenberg R. L. (1970). Location of aryl sulfatase in conidia and young mycelia of Neurospora crassa. J. Bacteriol. 104, 1254–1265. doi: 10.1128/jb.104.3.1254-1265.1970 16559101 PMC248285

[B141] Seidl V. (2008). Chitinases of filamentous fungi: a large group of diverse proteins with multiple physiological functions. Fungal Biol. Rev. 22, 36–42. doi: 10.1016/j.fbr.2008.03.002

[B142] Singh S. Madlala A. M. Prior B. A. (2003). Thermomyces lanuginosus: properties of strains and their hemicellulases. FEMS Microbiol. Rev. 27, 3–16. doi: 10.1016/S0168-6445(03)00018-4 12697339

[B143] Singh A. K. Mukhopadhyay M. (2012). Overview of fungal lipase: a review. Appl. Biochem. Biotechnol. 166, 486–520. doi: 10.1007/s12010-011-9444-3 22072143

[B144] Sinha R. Khare S. K. (2014). Protective role of salt in catalysis and maintaining structure of halophilic proteins against denaturation. Front. Microbiol. 5, 1–6. doi: 10.3389/fmicb.2014.00165 24782853 PMC3988381

[B145] Skliris N. Marsh R. Josey S. A. Good S. A. Liu C. Allan R. P. (2014). Salinity changes in the World Ocean since 1950 in relation to changing surface freshwater fluxes. Clim. Dynamics 43, 709–736. doi: 10.1007/s00382-014-2131-7

[B146] Souza P. M. D. Bittencourt M. L. D. A. Caprara C. C. Freitas M. D. Almeida R. P. C. D. Silveira D.. (2015). A biotechnology perspective of fungal proteases. Braz. J. Microbiol. 46, 337–346. doi: 10.1590/S1517-838246220140359 26273247 PMC4507524

[B147] Srivastava A. Saavedra D. E. M. Thomson B. García J. A. L. Zhao Z. Patrick W. M.. (2021). Enzyme promiscuity in natural environments: alkaline phosphatase in the ocean. ISME J. 15, 3375–3383. doi: 10.1038/s41396-021-01013-w 34050259 PMC8528806

[B148] Tarafdar J. C. Yadav R. S. Niwas R. (2002). Relative efficiency of fungal intra-and extracellular phosphatases and phytase. J. Plant Nutr. Soil Sci. 165, 17–19. doi: 10.1002/1522-2624(200202)165:1<17::AID-JPLN17>3.0.CO;2-C

[B149] Taylor J. D. Cunliffe M. (2016). Multi-year assessment of coastal planktonic fungi reveals environmental drivers of diversity and abundance. ISME J. 10, 2118–2128. doi: 10.1038/ismej.2016.24 26943623 PMC4989315

[B150] Thomson B. Wenley J. Currie K. Hepburn C. Herndl G. J. Baltar F. (2019). Resolving the paradox: continuous cell-free alkaline phosphatase activity despite high phosphate concentrations. Mar. Chem. 214, 1–6. doi: 10.1016/j.marchem.2019.103671

[B151] Traving S. J. Thygesen U. H. Riemann L. Stedmon C. A. (2015). A model of extracellular enzymes in free-living microbes: which strategy pays off? Appl. Environ. Microbiol. 81, 7385–7393. doi: 10.1128/AEM.02070-15 26253668 PMC4592861

[B152] Velicer G. J. (2003). Social strife in the microbial world. Trends Microbiol. 11, 330–337. doi: 10.1016/S0966-842X(03)00152-5 12875817

[B153] Vetter Y. Deming J. (1999). Growth rates of marine bacterial isolates on particulate organic substrates solubilized by freely released extracellular enzymes. Microbial Ecol. 37, 86–94. doi: 10.1007/s002489900133 9929397

[B154] Vetter Y. Deming J. Jumars P. A. Krieger-Brockett B. (1998). A predictive model of bacterial foraging by means of freely released extracellular enzymes. Microbial Ecol. 36, 75–92. doi: 10.1007/s002489900095 9622567

[B155] Wang Y. Barth D. Tamminen A. Wiebe M. G. (2016). Growth of marine fungi on polymeric substrates. BMC Biotechnol. 16, 1–9. doi: 10.1186/s12896-016-0233-5 26772742 PMC4715362

[B156] Wang L. Chi Z. Wang X. Liu Z. Li J. (2007). Diversity of lipase-producing yeasts from marine environments and oil hydrolysis by their crude enzymes. Ann. Microbiol. 57, 495–501. doi: 10.1007/BF03175345

[B157] Webster J. Weber R. (2007). Introduction to fungi (Cambridge: Cambridge University Press).

[B158] Weiss M. Abele U. Weckesser J. Welte W. Schiltz E. Schulz G. (1991). Molecular architecture and electrostatic properties of a bacterial porin. Science 254, 1627–1630. doi: 10.1126/science.1721242 1721242

[B159] Wetzel R. G. (1991). “Extracellular enzymatic interactions: storage, redistribution, and interspecific communication,” in Microbial enzymes in aquatic environments. Ed. Chróst R. J. (New York: Springer), 6–28.

[B160] Wheeler J. R. (1976). Fractionation by molecular weight of organic substances in Georgia coastal water. Limnol. Oceanogr. 21, 846–852. doi: 10.4319/lo.1976.21.6.0846

[B161] Wickerham L. J. (1939). A taxonomic study of Monilia albicans with special emphasis on morphology and morphological variation. J. Trop. Med. Hygiene 42, 174–179.

[B162] Wickerham L. J. (1951). Taxonomy of yeasts (Washington: US Department of Agriculture).

[B163] Zaccai G. (2004). The effect of water on protein dynamics. Philos. Trans. R. Soc. London. Ser. B: Biol. Sci. 359, 1269–1275. doi: 10.1098/rstb.2004.1503 15306381 PMC1693409

[B164] Zalar P. Kocuvan M. A. Plemenitaš A. Gunde-Cimerman N. (2005). Halophilic black yeasts colonize wood immersed in hypersaline water. Botanica Marina 48, 323–326. doi: 10.1515/BOT.2005.042

[B165] Ziervogel K. Arnosti C. (2008). Polysaccharide hydrolysis in aggregates and free enzyme activity in aggregate-free seawater from the north-eastern Gulf of Mexico. Environ. Microbiol. 10, 289–299. doi: 10.1111/j.1462-2920.2007.01451.x 18093165

